# Incorporation of Ellagic-Acid-Enriched Lignin into Hydrophilic Electrospun PDLLA/PVP Fibrous Scaffolds Imparts Remarkable Antioxidant and Cellular Redox Protection Properties

**DOI:** 10.3390/antiox15101224

**Published:** 2026-09-22

**Authors:** Rita Argenziano, Antonio Laezza, Luca Melotti, Roberta Sacchetto, Brigida Bochicchio, Antonietta Pepe, Samuele Giovando, Lucia Panzella, Alessandra Napolitano

**Affiliations:** 1 Department of Chemical Sciences, University of Naples “Federico II”, Via Cintia 4, 80126 Naples, Italy; rita.argenziano@unina.it (R.A.); panzella@unina.it (L.P.); 2 Department of Basic and Applied Sciences (DISBA), University of Basilicata, Via dell’Ateneo Lucano 10, 85100 Potenza, Italy; antonio.laezza@unibas.it or antonio.laezza@uniecampus.it (A.L.); brigida.bochicchio@unibas.it (B.B.); antonietta.pepe@unibas.it (A.P.); 3 Department of Theoretical and Applied Sciences (DiSTA), eCampus University, Via Isimbardi 10, 22060 Novedrate, Italy; 4 Department of Comparative Biomedicine and Food Science (BCA), University of Padova, Viale dell’Università 16, 35020 Legnaro, Italy; luca.melotti@unipd.it or l.melotti@unilink.it (L.M.); roberta.sacchetto@unipd.it (R.S.); 5 Department of Human Sciences, Link Campus University, Via del Casale di San Pio V 44, 00165 Rome, Italy; 6 Centro Ricerche per la Chimica Fine Srl for Silvateam Spa, Via Torre 7, 12080 San Michele Mondovì, Italy; sgiovando@silvateam.com

**Keywords:** lignin, ellagic acid, chestnut wood fiber, electrospinning, antioxidant scaffolds

## Abstract

A promising strategy to counteract reactive oxygen species (ROS) at cell–material interfaces, a condition that severely compromises cell viability and tissue regeneration, hinges on the incorporation of effective natural antioxidants into biocompatible polymeric scaffolds. Low- and high-molecular-weight phenolic compounds like ellagic acid (EA) and lignin can be easily obtained through green extraction methodologies from wood wastes, such as chestnut wood fiber (CWF), offering biocompatibility and intrinsic redox-protective properties. However, fabricating homogeneous lignin-based biomaterials remains challenging due to the inherent poor solubility properties of lignin. Herein, we report the fabrication of electrospun poly(d,l-lactide)/poly(vinylpyrrolidone) (PDLLA/PVP) mats loaded with an EA-enriched lignin (2% or 4% *w*/*w*) extracted from CWF via deep eutectic solvents (DES). The morphological and physicochemical properties of the scaffolds were characterized by scanning electron microscopy, attenuated total reflectance–Fourier transform infrared spectroscopy, and water contact angle. The EA–lignin-enriched mats exhibited remarkable antioxidant capacity, as evaluated by 2,2′-azino-bis(3-ethylbenzothiazoline-6-sulfonic acid (ABTS) and cupric reducing antioxidant capacity (CUPRAC) assays, coupled with a sustained and controlled release of EA under physiological conditions. Furthermore, the mats proved highly cytocompatible and capable of significantly mitigating intracellular ROS levels in both normal human dermal fibroblasts and mesenchymal stem cells subjected to oxidative insults. These findings demonstrate that the EA-enriched lignin can be successfully incorporated into electrospun scaffolds, yielding an effective antioxidant biomaterial for biomedical applications.

## 1. Introduction

Oxidative stress represents a critical factor affecting cell viability, proliferation, and tissue regeneration at the cell–material interface. High levels of reactive oxygen species (ROS) can severely damage cellular lipids, proteins, and DNA. This oxidative imbalance triggers chronic inflammatory responses and delays natural healing processes, highlighting an urgent need for functional biomaterials capable of controlling oxidative stress while maintaining cytocompatibility. In this context, the incorporation of natural compounds with marked antioxidant profiles into polymeric matrices has emerged as a promising strategy to confer intrinsic redox-protective properties to biomaterials [1].

Among sustainable biopolymers, lignin, which is the second most abundant carbon-based biopolymer after cellulose, has gained increasing attention in the biomedical sector due to its biocompatibility, wide availability, and intrinsic antioxidant activity. Notably, the biological performance of lignin is strongly dictated by its extraction method and natural sources [2,3]. Acting as a powerful antioxidant, lignin has been widely exploited as a green additive to improve the mechanical and functional properties of polymeric matrices for tissue engineering, drug delivery, diagnostics, food packaging, and environmental applications [1,4,5,6,7,8]. Given the growing interest in environmentally friendly and circular production processes, recent studies have focused on the exploitation of novel, sustainable sources of lignin, such as by-products and wastes from the agri-food and forestry industries. Among these, wood residues represent one of the cheapest and most abundant natural sources of lignin [9,10]. This biomass includes wastes from the industrial tannin industry, such as exhausted woods remaining after the hot-water extraction of commercial tannins. In particular, chestnut wood fiber (CWF), derived from exhausted chestnut wood, has been recently described as an easily available matrix with excellent antioxidant properties and pollutant adsorption capacity [11]. The main phenolic components of CWF consist of hydrolyzable ellagitannins, lignin, and low-molecular-weight phenolic derivatives, primarily ellagic acid (EA) [12]. Notably, EA is widely recognized for its potent anticancer, anti-inflammatory, antimicrobial, and cytoprotective effects. At the cellular level, EA not only acts as a direct scavenger of free radicals but also modulates key signaling pathways, upregulating endogenous antioxidant enzymes (such as superoxide dismutase and glutathione peroxidase) and mitigating oxidative insults at cell–material interfaces [13,14,15].

Due to its complex structure, high heterogeneity, and poor solubility, lignin exhibits limited compatibility with most hydrophobic polymer matrices and a strong tendency to undergo phase separation and severe molecular aggregation. To unlock its full biomedical potential and overcome this issue, advanced processing strategies using tailored, biocompatible polymeric blends are strictly required. In this regard, electrospinning has emerged as a powerful and versatile technique for the fabrication of fibrous scaffolds with controlled architecture, enabling the production of continuous fibers with diameters ranging from the nano- to the microscale. Owing their high surface-to-volume ratio and morphological similarity to the natural extracellular matrix (ECM), mats of electrospun fibers promote cell adhesion, proliferation, and migration.

Noticeable examples of biomedical applications of electrospun fibers loaded with lignin include polylactide–lignin nanofibers [16] and polycaprolactone (PCL)–lignin matrices [17] that have demonstrated remarkable cytoprotective activity and support toward neural cells, and polylactic acid (PLA)–lignin platforms showing great promise for cartilage regeneration [18]. Similarly, polyhydroxybutyrate (PHB)–lignin electrospun scaffolds [19] and collagen/chitosan matrices enriched with EA [20] have confirmed the potential of these bioactive components in counteracting oxidative damage. However, despite these outstanding bioactive properties, the fabrication of homogeneous, functional materials containing lignin remains a significant challenge.

Among biodegradable polyesters, poly(d,l-lactide) (PDLLA) is extensively investigated for scaffold fabrication due to its amorphous structure and low crystallinity, which result in enhanced organic solvent solubility and faster degradation kinetics compared to poly(l-lactide) (PLLA). To improve surface wettability and cell–matrix interactions, PDLLA is commonly blended with hydrophilic and biocompatible polymers. Poly(vinylpyrrolidone) (PVP) is especially attractive for this purpose due to its excellent water solubility, cytocompatibility, and capacity to enhance protein adsorption without compromising the mechanical integrity required for scaffold handling [21]. Crucially, blending PDLLA with a hydrophilic polymer like PVP offers a viable route to improve lignin dispersion within the volatile polymeric solution during electrospinning. Nonetheless, due to the viscoelastic perturbations induced by lignin, the formation of a “bead-on-a-string” (BOAS) morphology is frequently observed rather than smooth, bead-free fibers [22]. While traditionally considered a process defect, BOAS electrospun architectures have been reappraised as sophisticated, nanostructured carriers for therapeutic agents, owing to their high loading capacity of active compounds as well as to their controlled and sustained release [23,24].

In this work, we report the fabrication of e-spun PDLLA/PVP mats strategically designed to incorporate a lignin fraction enriched with a high content of EA, obtained via deep eutectic solvent (DES) extraction from CWF. By shifting the focus toward antioxidant functionality, this study investigates how the combination of PDLLA and PVP can overcome the traditional processing limitations of lignin. The designed scaffolds were evaluated for their ability to deliver a sustained release of EA in a physiological medium, together with their chemical antioxidant properties. Furthermore, their biological performance was evaluated through cytocompatibility assays and intracellular ROS measurements towards normal human dermal fibroblasts (NHDF) and mesenchymal stem cells (MSCs) to assess their efficiency in supporting cell viability while ensuring robust redox protection against oxidative insults.

## 2. Materials and Methods

Commercial-grade reagents and solvents were used without further purification. 1,1,3,3,3-Hexafluoro-2-propanol (HFP) was obtained from Iris Biotech GMBH (Marktredwitz, Germany). Dulbecco’s Modified Essential Medium (DMEM), antibiotic–antimicotic solution, fetal bovine serum (FBS), phosphate-buffered saline (PBS) solution, trypsin/EDTA solution, paraformaldehyde, polyvinylpyrrolidone (PVP, molecular weight: ~ 1300 KDa), Triton-X 100, 2,2′-azino-bis(3-ethylbenzothiazoline-6-sulfonic acid (ABTS), neocuproine, CuCl_2_, ammonium acetate, ethanol, methanol, choline chloride, tartaric acid, and H_2_O_2_ were obtained from Sigma-Merck (Darmstadt, Germany). Dimethylsulfoxide (DMSO) was purchased from Fluka (Seelze, Germany). Poly-d,l-lactic acid (PDLLA, molecular weight: 126 kDa) was obtained from FormFutura (Nijmegen, The Netherlands). AlamarBlue™ Cell Viability Reagent, Hoechst 33342, Tetramethylrhodamine B isothiocyanate-conjugated (TRITC) phalloidin, and 5-chloromethyl-2′,7′-dichlorodrofluorescein diacetate (CM-H2DCFDA) were obtained from Thermofisher Scientific (Waltham, MA, USA). Silvateam (S. Michele Mondovì, Cuneo, Italy) provided CWF that is not commercially available. This CWF was obtained from exhausted chestnut wood after drying in an oven overnight at 60 °C followed by milling to obtain <250 µm particles. Fluorescent readings for cell culture experiments were recorded using a Varioskan Microplate reader (Thermofisher Scientific, Waltham, MA, USA). Cells were visualized using a fluorescence microscope Thunder Imager (Leica, Wetzlar, Germany).

UV–Vis spectra were recorded using a Jasco (Cremella, Lecco, Italy) V-730 spectrophotometer.

HPLC analyses were performed on an instrument equipped with a UV–Vis detector (Agilent, Cernusco sul Naviglio, Milan, Italy); a Phenomenex (Castel Maggiore, Bologna, Italy) Sphereclone ODS column (250 × 4.60 mm, 5 µm) was used, and a gradient elution was performed using 0.1% formic acid (solvent A)/methanol (solvent B): 5% B, 0–10 min; from 5 to 80% B, 10–47.5 min. The flow rate was 1.0 mL/min, and the detection wavelength was 360 nm.

### 2.1. Preparation of EA-Enriched Lignin

A previously described method [25] was adopted with slight modifications. Briefly, choline chloride and tartaric acid were combined in a 1:2 molar ratio and were stirred at 50 °C until a clear and uniform solution was obtained. The resulting DES was stored at room temperature in the absence of light, and no crystallization phenomena were detected throughout its utilization period. Subsequently, CWF (10 g) was dissolved in 100 g of the DES containing 20% *w*/*w* water and stirred at 50 °C for 90 min. After cooling to room temperature, the suspension was centrifuged at 7000 rpm for 10 min at 4 °C to separate the insoluble fraction. The resulting solution was slowly poured into 1 L of 1% KCl and stored at 4 °C to promote precipitation. The material was then collected by centrifugation (7000 rpm, 20 min, 4 °C), washed with distilled 1% KCl solution, and finally freeze-dried, yielding approximately 28% *w*/*w* of extracted material (EA-lignin).

### 2.2. Preparation of Hazelnut and Chestnut Lignin Mixed with EA–Lysine Salts

Lignin was extracted from hazelnut and chestnut biomasses according to a previously reported procedure [3]. To prepare the ellagic acid–lysine salt (EA–Lys), EA and l-lysine were reacted at a 1:4 molar ratio in distilled water under continuous stirring for 30 min, and the resulting solution was lyophilized. Subsequently, the EA–Lys salt and the extracted lignin (from hazelnut or chestnut) were mixed in a ball mill for 15 min at a frequency of 50 Hz.

### 2.3. Electrospinning

Electrospinning was performed on a Linari Engineering Gamma-High-Voltage generator electrospinning system. The polymer mixtures were loaded into a 10 mL glass syringe with a 20 G stainless-steel needle and then electrospun at 17 kV with a flow rate of 1.0 mL/h of the pump. The target was a round copper plate with 90 mm diameter coated with aluminum foil, and the distance between the collector and the needle was set to 19 cm. The composition of the solutions used for electrospinning is listed in Table 1.

The preparation of electrospinning solution/suspension was performed according to the following general protocol:

EA-lignin, when present, was suspended in DMSO and allowed to stand for 2 h at room temperature with stirring. During this time, the suspension was sonicated hourly for 15 min. Subsequently, the prepared EA-lignin suspension was added to solid PVP, and the mixture was stirred overnight at 37 °C. In parallel, PDLLA was suspended in HFP and left under stirring overnight at 37 °C. Finally, the two resulting samples were combined, stirred, sonicated for 15 min, and electrospun.

Specifically, for mats of EA-lignin2@PDLLA/PVP and EA-lignin4@PDLLA/PVP, EA-lignin (6.0 or 12.0 mg, respectively) was suspended in DMSO (600 μL) and stirred at room temperature for 2 h and sonicated. Subsequently, the suspension was added to solid PVP (45.0 mg), and the mixture was kept under magnetic stirring overnight at 37 °C. In parallel, PDLLA (300.0 mg) was dissolved in HFP (2.40 mL) and kept under magnetic stirring overnight at 37 °C. Finally, the suspension containing EA-lignin and PVP was combined with the PDLLA solution, stirred, and sonicated.

For control scaffolds such as PDLLA/PVP, PVP (45 mg) was dissolved in DMSO (600 μL) and stirred at room temperature for 2 h, sonicated, and kept under magnetic stirring overnight at 37 °C. Subsequently, HFP (2.40 mL) was added, and the solution was treated as above. For the control scaffold PDLLA, 300 mg of the polyester was dissolved in HFP (2.40 mL) and kept under magnetic stirring overnight at 37 °C. Subsequently, DMSO (600 μL) was added, and the solution was treated as above.

### 2.4. Morphology Characterization of Scanning Electron Microscopy (SEM) Images

SEM images were acquired with a voltage of 20 kV and different magnifications, after gold sputter-coating on a FEI Quanta 400 high-resolution field emission scanning electron microscope (ESEM) instrument. Samples collected after incubation were also gold sputter-coated and analyzed using a Philips-FEI ESEM XL30 (FEI, Eindhoven, The Netherlands) scanning electron microscope operating at 30 kV.

The average fiber diameter was determined manually using ImageJ 1.54g software by measuring individual fibers from SEM images acquired at three different regions of each scaffold. From each region, 50 individual fibers were measured for a total of 150. Manual measurements were preferred over automated image-analysis procedures, such as the DiameterJ plugin, because the bead-on-string (BOAS) morphology could lead to an overestimation of the actual fiber diameter. Digital porosity (DP) was determined by Image J analysis from SEM images of three different positions of each type of scaffold. The application of contrast enhancement and a filter for noise reduction was performed before thresholding. Then, the SEM images were converted to binary areas of pores and fibrous moieties in black and white by adjusting threshold grayscales with the Otsu method. For apparent roughness calculation, the acquired SEM images were converted to 32 bit and scaled prior to analysis. Then, the ImageJ plugin, “SurfCharJ” was used to measure the roughness profiles of root-mean-square roughness (Rq). Each measurement was reiterated on three SEM images per scaffold type, and data were expressed as the mean ± SD (*n* = 3).

### 2.5. Attenuated Total Reflectance Fourier Transform Infrared (ATR-FTIR) Spectroscopy

ATR-FTIR spectra were acquired on a J-460 (Jasco Europe Srl, Cremella, Lecco, Italy) equipped with an ATR PRO ONE single-reflection ATR accessory using a single crystal diamond ATR prism. Spectra were acquired in the region from 4000 to 400 cm^−1^ with a spectral resolution of 2 cm^−1^ and 256 scans. Background spectra were recorded each time and then subtracted from the sample spectra.

### 2.6. Static Water Contact Angle (SWCA)

Surface wettability of the e-spun scaffolds was evaluated by static water contact angle (SWCA) measurements. A droplet of ultrapure water (2 μL) was deposited onto the scaffold surface using an automated syringe system. All measurements were performed at room temperature. The contact angle was recorded 10 s after droplet deposition to standardize the measurement and minimize the influence of rapid absorption phenomena. Results were calculated from measurements obtained from four different areas of the same scaffold. The contact angle was measured using ImageJ 1.54g software supplied with the Low-Bond Axisymmetric Drop Shape Analysis method in the Drop Analysis plug-in.

### 2.7. In Vitro Antioxidant Assays of E-Spun Mats

Sections of the all-prepared e-spun mats with a total surface area of 4 cm^2^ (corresponding to 5 mg of material) were placed at the bottom of vials containing various volumes of the CUPRAC reagent, yielding final scaffold-to-solution concentrations of 0.0025 mg/mL, 0.00125 mg/mL, and 0.00085 mg/mL. The CUPRAC solution was prepared by mixing CuCl_2_ (10 mM), neocuproine (7.5 mM), ammonium acetate buffer (1 M at pH 7.0), and water in a ratio of 1:1:1:0.9 *v*/*v*, in that order. The absorbance of each solution was periodically monitored at 450 nm at fixed time intervals (20, 40, and 60 min). All experiments were performed in triplicate, and the results were expressed as Trolox equivalents. The time-dependent radical scavenging kinetics were further investigated via the ABTS assay, adapting the same volume and concentration parameters from the previously described CUPRAC protocol. The ABTS radical cation (ABTS^•+^) was generated by dissolving ABTS (7.6 mg) in water and reacting it with potassium persulfate (K_2_S_2_O_8_, 1.3 mg). The mixture was kept in the dark at 27 °C for 16 h to allow complete formation of the ABTS•^+^ radical, as confirmed by a stable absorbance at 734 nm. Prior to use, the ABTS•^+^ solution was diluted with PBS (1×), until an initial absorbance of 0.70 at 734 nm was reached.

### 2.8. Ellagic Acid Release Kinetics

EA-lignin4@PDLLA/PVP and EA-lignin2@PDLLA/PVP were cut into square sections (2 × 2 cm^2^). Each sample was dipped in a known volume of PBS (1×), to achieve a *w*/*v* ratio of 10 mg/mL. The samples were incubated under controlled conditions at 37 °C and the medium replaced every 24 h. The release of EA was monitored every 24 h for 6 d.

For gravimetric mass loss measurement, the samples were weighted before (*Mb*) and after incubation (*Ma*). The mass loss (*ML*) was calculated according to the following equation:

$$ML\left(\%\right)=\frac{Mb-Ma}{Mb}\times 100$$

### 2.9. ABTS Assay on the Release Medium of EA-Lignin4@PDLLA/PVP

The ABTS radical cation (ABTS^•+^) was generated as described in Section 2.7. The released sample obtained from EA-lignin4@PDLLA/PVP after 24 h dipping in PBS as described above was collected, dried under vacuum, and redissolved in methanol at known concentrations. Subsequently, the resulting solution was added at different volumes (25, 50, 100 μL) to the prepared ABTS•^+^ solution, and the decrease in absorbance at 734 nm was monitored after 10 min.

### 2.10. Cell Culture and Cell Viability Assays

Normal human dermal fibroblasts (NHDF) and mesenchymal stem cells derived from adipose tissue (MSCs) were isolated and characterized as previously described [26,27]. Cells were cultured in low-glucose DMEM (1000 mg/L), supplemented with 15% (*w*/*v*) FBS along with a solution of antibiotic–antimycotic (100 U/mL penicillin, 100 mg/mL streptomycin, and 25 μg/mL amphotericin B). Cells were kept in a humified 5% (*v*/*v*) CO_2_ atmosphere at 37 °C. Cells were refreshed with cell culture medium every 2–3 d and were used for experiments at passages 3–6. At sub-confluence (85%), cells were detached using trypsin/EDTA solution (2.5 g/L/0.2 g/L) and seeded accordingly to perform experiments. Prior to experiments, thin films were cut using sterilized scissors into pieces of 1 cm per side; afterwards, they were sterilized under UV-light exposure for 30 min each side. For cytocompatibility, cells were seeded dropwise at a final concentration of 2.5 × 10^4^ cells/mat onto the thin film and left to incubate for 1 h at 37 °C in a humified 5% (*v*/*v*) CO_2_ incubator to allow cell attachment. At the end of the incubation period, cell culture medium was added to each well. After 1, 3, and 5 d in culture, the cytocompatibility of the material was evaluated using the alamarBlue™ Cell Viability Reagent; the alamarBlue™ working solution was used at a final concentration of 10% in complete cell culture medium. Briefly, samples were washed twice with PBS, and then 500 µL of alamarBlue™ working solution was added to each well; samples were incubated for 4 h in a humified chamber (5% CO_2_, *v*/*v*) at 37 °C in the dark. Then, 100 µL of supernatant solution was aliquoted into a 96-well culture plate for a total of five times per sample. Fluorescence was measured using a microplate reader (Varioskan) at λ = 544/590 nm (ex/em). The mat without cells was used as blank sample, and cells cultured onto cell culture plastic were considered as positive control. The experiments were repeated at least three times. When indicated, after 1 day, cells were exposed to 200 µM H_2_O_2_ to induce oxidative stress for 2 h and kept in an incubator at 37 °C (5% CO_2_). At the end of incubation, cell viability was assessed as previously described. Data were expressed as % of viability and normalized on each control condition (i.e., cells grown onto mats without oxidative stimuli).

### 2.11. Cell Morphology

Cell morphology was visualized by staining cells with fluorescent dyes for cytoskeleton (phalloidin) and nucleus (Hoechst 33342). Cells were grown onto mats as previously described and images were acquired using a fluorescence microscope after 1, 3, and 5 d in culture. At each time points, mats were washed three times with PBS and fixed with 4% paraformaldehyde for 15 min at room temperature. After fixation and several washes using PBS, samples were permeabilized with 0.5% (*v*/*v*) Triton X-100 in PBS for 10 min, and then stained with 50 µg/mL TRITC-conjugated phalloidin and Hoechst, to stain f-actin cytoskeleton and nuclei, respectively. Images were acquired using the Thunder Imager fluorescence microscope.

### 2.12. ROS Levels Quantification

ROS levels in both NDHF and MSCs cultured in an oxidative environment were assessed using the fluorescent probe CM-H2DCFDA following the manufacturer’s instructions with modifications. Cells were seeded onto mats at a density of 2.5 × 10^4^; after 24 h in culture in complete cell culture medium, cells were washed using a solution of 10 mM glucose in PBS. Afterwards, 5 μM M CM-H2DCFDA dye was added to each sample and incubated for 30 min in the dark at 37 °C. Thereafter, samples were washed with 10 mM glucose-PBS; eventually, samples were challenged with 200 μM H_2_O_2_ for 2 h. Fluorescent positivity was estimated using a microscope (Thunder Imager); images were acquired using the same gain settings and exposure for all samples. Fluorescence was quantified using the ImageJ image analyzer software.

### 2.13. Statistical Analysis of Data

Statistical analyses were performed using the software GraphPad Prism 9.0.0 (GraphPad Software, San Diego, CA, USA). Data were compared using the one-way ANOVA statistical test and Tukey’s ‘post hoc’ test. Differences were considered statistically significant with values of *p* < 0.05. SWCA and antioxidant activity data were expressed as mean ± standard error (SD). Metabolic activity data were expressed as mean ± standard error of the mean (SEM).

## 3. Results

### 3.1. Preparation of EA-Enriched Lignin

EA-enriched lignin was recovered employing DES as green and ecofriendly extraction solvents following a protocol described in our previous studies [25]. Briefly, lignin was extracted using chlorine chloride and tartaric acid (1:2 mol/mol ratio) for 90 min at 50 °C. After the addition of 1% *w*/*w* KCl, lignin was simply recovered by precipitation followed by centrifugation and extensive washings with the KCl solution. As already reported, the mild extraction conditions of this method allow one to obtain a peculiar lignin enriched in ellagic acid (33% *w*/*w*), exhibiting remarkable antioxidant properties.

### 3.2. Preparation of E-Spun Mats with EA-Enriched Lignin

With the aim of obtaining functional hybrid materials with antioxidant activity, PDLLA has been selected as a polymer matrix, due to its wide application as a biomaterial, and the EA-lignin sample, prepared as described above, was incorporated within it. Preliminary attempts regarding process conditions and chemical composition were made to obtain a stable electrospinning process, before selection of the best composition of the mixture (see Table 1, Section 2).

PDLLA is known to be easily electrospun, giving rise to homogeneous scaffolds; however, its hydrophobicity represents one of the major limitations for most biological applications. To overcome this drawback, PVP was taken as a suitable additive in this study. PVP is highly water-soluble and was introduced to enhance matrix hydrophilicity and promote controlled porosity and consequent release upon exposure to aqueous media. Pure PVP films were not considered suitable due to their rapid dissolution in aqueous media, which would result in burst rather than sustained release.

In search of a suitable solvent system, HFP was selected based on its current use for dissolving and electrospinning of PDLLA [28], while DMSO proved suitable for lignin [29]. Both solvents were employed in the production of composite e-spun materials containing PVP and poly-d,l-lactide [30,31,32]. Under these conditions, the concentration of PDLLA was 10% *w*/*v*, while the concentrations of PVP and EA-lignin (relative to PDLLA) were 15% *w*/*w* for PVP and 2% and 4% *w*/*w* for EA-lignin, respectively.

The morphological properties of the obtained scaffolds were then evaluated by scanning electron microscopy (SEM). All the e-spun mats showed randomly oriented fibers in a three-dimensional microstructure enriched with interconnected pores. At lignin concentrations of 2% (EA-lignin2@PDLLA/PVP) and 4% *w*/*w* (EA-lignin4@PDLLA/PVP), a BOAS morphology and fiber fusion were observed (Figure S1). This feature could be optimal for drug loading and sustained release. The beads, indeed, can not only completely encapsulate water-soluble drugs or solid particles, but their degradation would give rise to a sustained release [33]. BOAS morphology is generally associated with inadequate chain entanglement, which could result from low viscosity or insufficient polymer concentration. [34,35]. The presence of lignin featuring a complex structure of aromatic units connected through diverse C-C and C-O bonds may have favored the formation of beads, thereby reducing chain entanglement [36]. Moreover, the presence of DMSO, which is characterized by a lower surface tension with respect to HFP, could have hampered complete drying of the polymer jet reaching the collector, leading to the formation of beaded fibers, as apparent in the case of PDLLA/PVP and PDLLA mats prepared without lignin addition (Figure S1) [37]. In addition to sample properties, the morphology of the mats is also affected by the process conditions. BOAS morphology, indeed, generally occurs when the surface tension of the solution overcomes the electrostatic forces [22,38]. Yet, how each of these parameters contribute to the resulting matrix is difficult to assess.

### 3.3. Chemical Composition of E-Spun Mats

To obtain insights into the chemical composition, successful blending, and incorporation of the EA-lignin within the e-spun mats, ATR-FTIR investigation was carried out.

Initially, the pristine polymeric components and the unloaded matrix were characterized to establish a reliable baseline (Figure S2). PDLLA/PVP (red curve), showed a broad band in the region 3700–3100 cm^−1^ (black circle), also observed in the spectrum of pristine PVP, associated with the O-H stretching band of absorbed water, highlighting the hygroscopic nature of PVP [39]. Bands at ~2998 and ~2948 cm^−1^ (red circle) are associated with aliphatic C−H stretching vibrations derived both from pristine PVP and PDLLA. Additionally, intense bands at ~1754 (light green) and ~1658 cm^−1^ (light blue) are associated with the PDLLA ester and PVP lactam C=O stretching vibration, respectively. These characteristic bands are clearly observed in PDLLA and pristine PVP spectra. Moreover, the bending vibration bands of aliphatic C-H in methylene groups are visible at ~1451 cm^−1^ and in the 1492–1421 cm^−1^ region (grey circle) for PDLLA/PVP and pristine PVP, respectively. The bending vibration band of C-H in the methyl group falls at the same value for PDLLA/PVP and pristine PDLLA.

In parallel, the pure EA-lignin powder was evaluated as a reference. The spectrum of EA-lignin powder (Figure S3) showed a shoulder at 3550 cm^−1^ (blue circle) that is characteristic of EA and a broad band in the region between 3500 and 3090 cm^−1^ (black circle) associated with the O-H stretching. Moreover, the peaks associated with the C-H stretching (red circle) are also visible at 2916 and 2849 cm^−1^ [25]. At ~1732, ~1715, and ~1700 cm^−1^ (green circle), the two peaks can be associated with the C=O of esters, non-conjugated ketones, and carbonyls in lignin [40] and ellagic acid, respectively [41]. Bands at ~1614, ~1582, and ~1510 cm^−1^ (orange circle) corresponding to the aromatic C=C stretching vibrations, as well as the band at ~1451 cm^−1^ (grey circle) assigned to the asymmetric vibrations and deformation of the C–H in methylene and methyl groups, are also visible [42]. Moreover, the presence of syringyl (1330 and 1110 cm^−1^, yellow circles) and guaiacyl units (1260, 1207, and 1053 cm^−1^, purple circles) can be observed [43,44].

The ATR-FTIR spectra of the hybrid scaffolds loaded with different amounts of filler, namely EA-lignin2@PDLLA/PVP (purple curve) and EA-lignin4@PDLLA/PVP (blue curve), were compared in the magnified 1900–500 cm^−1^ region (Figure S4). To allow a meaningful comparison, the absorbance profiles were normalized with respect to the pristine PDLLA ester C=O stretching band at 1754 cm^−1^. Moving from EA-lignin2@PDLLA/PVP to EA-lignin4@PDLLA/PVP, the presence of a shoulder peak at ∼1717 cm^−1^ (red line) becomes evident. This feature perfectly matches the carbonyl stretching region identified in the pure EA-lignin powder, serving as a direct marker for filler presence [45]. A relative increase in absorbance intensity is detected at 1652 cm^−1^ when comparing EA-lignin2@PDLLA/PVP to EA-lignin4@PDLLA/PVP (green line). Since the nominal PVP content is kept constant across the blend formulations, this local intensity enhancement is physically attributed to the overlapping contribution of the intense aromatic ring skeleton vibrations C=C stretching at 1614–1582 cm^−1^ belonging to the EA-lignin fraction. Importantly, the spectral profiles in the 1451 cm^−1^ region (C-H deformations) and the 1210 cm^−1^ region (guaiacyl unit range) do not follow a strictly monotonic trend with filler loading. This is because these specific zones are heavily dominated by the massive, underlying absorption bands of the hosting PDLLA matrix (such as the main C-O-C asymmetric stretching). Ultimately, the fact that the primary diagnostic bands of both PDLLA and PVP remain perfectly resolved and unshifted upon filler loading strongly implies that the incorporation of up to 4% *w*/*w* of EA-rich lignin does not alter the structural integrity or induce chemical degradation in the hosting polymeric matrix. These findings seem to confirm the suitability of the processing method for preparing stable hybrid mats. The proposed frequency assignments are summarized in Table 2.

### 3.4. SWCA Measurement

To evaluate the effects of incorporation of EA-lignin on surface wettability, SWCA measurements were performed on the e-spun mats with different lignin loadings (Figure S5), affording the values reported in Table 3. The control sample PDLLA exhibited a contact angle of 115 ± 2°, confirming the intrinsic hydrophobic character of the polyester. In contrast, the addition of PVP markedly reduced the contact angle in the control scaffold PDLLA/PVP with a value of 75 ± 1°, indicating that this polymer significantly reduced the hydrophobicity of the matrix.

When EA-lignin was incorporated at 2% *w*/*w* (EA-lignin2@PDLLA/PVP), the SWCA increased from 75 ± 1° for PDLLA/PVP to 98 ± 1°, indicating a reduction in surface wettability. A much more pronounced increase was observed at 4% *w*/*w* EA-lignin (EA-lignin4@PDLLA/PVP), for which the SWCA reached 141 ± 1°. This concentration-dependent behavior suggests that the EA-lignin fraction contributes to modifying the surface properties of the scaffold. Lignin is intrinsically amphiphilic, while ellagic acid, although containing several polar hydroxyl groups, exhibits very low water solubility because of its extended aromatic structure and strong intermolecular interactions. Therefore, increasing the EA-lignin loading may contribute to decreasing the effective wettability of the fibrous surface. However, the marked increase in SWCA observed for the 4% formulation cannot be explained by chemical composition alone and is likely related to a cooperative effect between surface composition and a different surface texturing of the electrospun scaffold, a Cassie–Baxter state, where air pockets imprison water droplets, artificially increasing the apparent contact angle, as already reported [46].

Quantitative analysis of the SEM images provided further insight into this behavior (Table 4). Incorporation of EA-lignin markedly increased the digital porosity (DP) of the scaffolds, from 28.3 ± 5.0% for PDLLA/PVP to 53.8 ± 3.4% and 52.3 ± 6.6% for EA-lignin2@PDLLA/PVP and EA-lignin4@PDLLA/PVP, respectively. In contrast, the apparent root-mean-square roughness (*Rq*) remained comparable among the formulations, with values of 0.48 ± 0.02, 0.49 ± 0.03, and 0.53 ± 0.04 AU for PDLLA/PVP, EA-lignin2@PDLLA/PVP, and EA-lignin4@PDLLA/PVP, respectively. Thus, the pronounced increase in SWCA from the 2% to the 4% formulation cannot be attributed to a further increase in either average porosity or apparent roughness.

SEM analysis also provided information on fiber diameter. Fiber diameter distributions (Figures S6 and S7) were approximately normal, with mean values of 0.43 ± 0.10, 0.44 ± 0.07, 0.40 ± 0.08 μm, and 0.37 ± 0.07 for EA-lignin2@PDLLA/PVP, EA-lignin4@PDLLA/PVP, and PDLLA/PVP, and PDLLA, respectively. The addition of PVP induced a slight increase in fiber diameter, typically associated with an enhancement of the viscosity of polymeric mixtures [47]. A further modest increase was observed upon incorporation of EA-lignin at both 2% and 4% (Figures S6 and S7). A similar trend was observed in PLA/lignin electrospun composites [48].

Overall, both EA-lignin-containing scaffolds exhibit a highly porous bead-on-string architecture that can favor air retention within the fibrous network at the water/scaffold interface. Increasing the EA-lignin concentration from 2% to 4%, while keeping the PDLLA and PVP contents constant, doubles the amount of the EA-rich aromatic filler incorporated into this porous architecture. The higher contribution of EA-lignin in the 4% formulation is also supported by ATR-FTIR analysis, which shows a more evident contribution of characteristic EA-lignin bands at the higher loading. We therefore propose that the combination of a less wettable surface composition and a highly porous fibrous architecture favors the stabilization of a Cassie–Baxter-like wetting regime, in which the water droplet initially rests on a composite interface consisting of solid fibers and entrapped air. Such a cooperative effect may substantially amplify the apparent contact angle even in the absence of a measurable increase in average surface roughness [48].

It should also be noted that the SWCA was measured 10 s after droplet deposition. Accordingly, the value of 141 ± 1° should be interpreted as a short-time apparent contact angle reflecting an initial kinetic barrier to wetting, rather than as an equilibrium contact angle. Upon prolonged exposure to aqueous media, water can progressively penetrate the porous scaffold, facilitated by the hydrophilic PVP-containing phase, allowing release of the encapsulated EA-enriched lignin components and preservation of the antioxidant functionality of the material.

### 3.5. Time-Dependent Antioxidant Properties of E-Spun Mats

To evaluate the functional performance of the e-spun mats, the antioxidant properties were monitored over time (20, 40, and 60 min) at physiological pH (pH 7.0) using commonly adopted chemical assays such as ABTS and CUPRAC. Experiments were conducted by immersing 4 cm^2^ pieces of the e-spun mats into various volumes, yielding final scaffold-to-solution concentrations of 0.0025 mg/mL, 0.00125 mg/mL, and 0.00085 mg/mL. These concentrations were selected for the ABTS and CUPRAC assays to prevent immediate quenching of the radical species and to operate within the linear sensitivity range of the antioxidant method. The kinetic profiles for the ABTS radical scavenging activity of EA-lignin2@PDLLA/PVP and EA-lignin4@PDLLA/PVP and of PDLLA/PVP are reported in Figure S8. In this assay, the radical cation ABTS^•+^ is generated by oxidation of ABTS with potassium persulfate, showing a characteristic absorption band at 734 nm. Monitoring the decrease in this absorbance over time allows for the evaluation of the antioxidant capacity of the e-spun mats. At all tested solution concentrations, the PDLLA/PVP control matrix induced a low but still detectable ABTS^•+^ consumption that did not exceed 30–50% even after 60 min of incubation, which is presumably ascribed to physical entrapment mechanisms. In contrast, EA-lignin incorporation provided a remarkable, dose-dependent radical scavenging capacity. As shown in Figure 1A, at the highest solution concentration of 0.0025 mg/mL, the EA-lignin2@PDLLA/PVP mat steadily overtook the control, reaching a consumption of 68% at 60 min, while the EA-lignin4@PDLLA/PVP scaffold achieved 80% of consumption at the same time, demonstrating a superior antioxidant capacity, as expected due to the higher amount of EA-lignin incorporated. It is worth noting that while the high SWCA of the EA-lignin4@PDLLA/PVP values reflects a temporary, non-wetting kinetic barrier at the interface, it does not hinder the antioxidant performance of the mats in aqueous media. In fact, upon prolonged immersion, the underlying hydrophilic PVP channels facilitate water swelling and matrix penetration, allowing the encapsulated EA-enriched lignin to fully exert its antioxidant properties. Importantly, higher filler loadings (e.g., 6% *w*/*w*) were investigated during preliminary screening but systematically excluded from the final study since higher EA-lignin loading into the e-spun mats caused detrimental surface wetting limitations and a subsequent drop in antioxidant release efficiency, identifying the 4% *w*/*w* formulation as the optimal compromise between surface morphology and functional performance. A similar dose-dependent trend was observed in the CUPRAC assay at 60 min (Figure S9 and Figure 1B), confirming copper-ion reducing power upon increasing both the EA-lignin content and the overall mat concentration in the medium. Furthermore, the fact that both the ABTS (which monitors single-electron transfer and hydrogen-atom transfer pathways) and the CUPRAC (probing copper-ion reducing capacity by single electron transfer only) assays yielded highly reproducible and analogous dose–response results confirms the EA-lignin4@PDLLA/PVP mat as an optimal potential active delivery platform. However, a closer inspection of the CUPRAC data reveals a less pronounced performance at lower testing concentrations (0.00125 and 0.00085 mg/mL). In fact, under these diluted conditions, only the highest loaded scaffold (EA-lignin4@PDLLA/PVP) exhibited higher antioxidant ability compared to the control PDLLA/PVP. Because the CUPRAC electron-transfer mechanism appears less sensitive to the control compared to the highly responsive ABTS assay, subsequent kinetic release monitoring and advanced antioxidant investigations were carried out exclusively utilizing the ABTS assay.

### 3.6. In Vitro Release Kinetics of Ellagic Acid from the E-Spun Mats

The effective release of EA from the prepared mats (EA-lignin2@PDLLA/PVP and EA-lignin4@PDLLA/PVP) was evaluated under biomimetic conditions in 1× PBS at 37 °C. The e-spun mats were immersed in the buffer solution at a sample-to-volume concentration of 10 mg/mL and incubated under static conditions. The concentration range was chosen to ensure that the released species would reach a concentration above the detection limit, thus allowing straightforward UV–Vis spectrophotometric monitoring over time. To closely mimic a realistic in vivo microenvironment—such as physiological fluid turnover, capillary circulation, or wound exudate clearance—the release medium was completely collected and replaced with fresh PBS every 24 h over 6 d. This dynamic, non-cumulative sampling strategy ensures that the system does not reach an artificial thermodynamic saturation, effectively profiling the daily continuous delivery capacity of the scaffolds. The release profile was monitored by UV–Vis spectroscopy by measuring the characteristic absorbance of EA moieties at 360 nm. As illustrated in Figure 2, a pronounced release was observed within the first 24 h for all investigated samples. In particular, the highest absorbance value was obtained for the EA-lignin4@PDLLA/PVP scaffold compared to EA-lignin2@PDLLA/PVP. Notably, at longer incubation time (Figure 2), the EA-lignin-loaded mats exhibited a lower but constant release rate. These release findings are in perfect agreement with the trend obtained from the chemical radical scavenging assays, suggesting that the superior performance of the EA-lignin4@PDLLA/PVP mat could be driven by its ability to ensure a higher, sustained release of the active antioxidant filler over time. Evaluation of the cumulative absorbance showed, as expected, a steep increase over the first 3 d and then a smoothing down, reaching a plateau over the observation period (Figure S10).

To obtain further information, the UV–Vis spectrum of the species released from EA-lignin4@PDLLA/PVP was recorded. As shown in Figure S11, a distinct absorption maximum at 360 nm was observed, consistent with that of EA (greenish line), whereas no absorption maximum was detected for the PDLLA/PVP control sample. Furthermore, to quantify the amount of EA released, a calibration curve of ellagic acid was constructed. The concentration of EA released after 24 h for the sample EA-lignin4@PDLLA/PVP was determined to be 14 µM. However, a possible limitation of UV–Vis analysis is the interference from other compounds present in the system, particularly lignin, which may also absorb at 360 nm and thus artificially increase the measured absorbance. To obtain a more accurate quantification, the release solution collected after 24 h was further analyzed by high-performance liquid chromatography (HPLC), yielding an ellagic acid concentration of 12 µM, which is in good agreement with the value estimated by UV–Vis spectroscopy. The quantitative total release of EA monitored over 6 days via UV–Vis spectroscopy (λ=360 nm) reached cumulative values of 50.47 ± 0.01% for EA-lignin4@PDLLA/PVP mats and 45.4 ± 0.2% for EA-lignin2@PDLLA/PVP mats relative to the total EA content in the mats.

To further evaluate scaffold stability under aqueous conditions, mass loss was determined after 6 days of incubation in PBS. Pure PDLLA scaffold exhibited only a negligible mass loss (0.33 ± 0.06%), whereas incorporation of PVP increased the value to 3.1 ± 2.9%. Higher mass losses were observed for EA-lignin2@PDLLA/PVP (6.0 ± 3.4%) and EA-lignin4@PDLLA/PVP (8.8 ± 2.9%). The observed trend is consistent with increasing amounts of water-accessible and potentially releasable components within the composite scaffolds. In particular, partial dissolution of PVP together with release of the EA-enriched lignin fraction may contribute to the measured mass loss, while the PDLLA component provides structural persistence. SEM analysis after incubation showed a partial rearrangement of the fibrous architecture, including local fiber swelling and fusion, but without complete loss of the interconnected fibrous network. These morphological changes are consistent with water penetration into the PDLLA/PVP matrix and partial dissolution of its water-soluble fraction. Importantly, despite these changes and the concomitant mass loss, the scaffold remained macroscopically intact throughout the investigated incubation period, indicating that the PDLLA-rich phase was sufficient to preserve the overall structural framework (Figure S12).

Moreover, to assess the role of lignin as a carrier for EA, control formulations were prepared as physical mixtures. Specifically, lignins extracted from hazelnut and chestnut, as already reported before [3], were combined with EA–lysine salt via ball milling at the same *w*/*w* ratio as the EA-lignin recovered from CWF. Briefly, to prepare the salt, EA and l-lysine were mixed at a 1:4 molar ratio in water for 30 min, followed by lyophilization to yield the dry salt. Subsequently, the EA–lysine salt was mixed with hazelnut or chestnut lignin in a ball mill for 15 min at 50 Hz to enhance the dispersion of EA within the lignin, thereby improving the aqueous solubility of EA. As shown in Figure S12, under the same experimental conditions described above, significantly lower release was observed at all times. Notably, chestnut lignin intrinsically contained ca. 1% EA, as previously reported [3], and exhibited a slightly higher absorbance during the release assays compared to hazelnut lignin. Overall, these results demonstrate that when an equivalent amount of pure EA (30 wt%) was physically mixed with lignin, the release remained significantly lower than that of EA-enriched lignins, as observed for both chestnut lignin and, most notably, the EA-lignin recovered from CWF. In fact, it should be highlighted that DES treatment promotes the cleavage of hydrolyzable tannins, thereby enriching the matrix with EA in a highly integrated manner. This confirms that the DES-mediated process creates a structurally synergistic EA–lignin system that performs vastly better than a simple physical mixture of its individual components. A possible explanation lies in the molecular dispersion achieved during DES treatment. Unlike physical mixing, which could leave hydrophobic EA aggregates within the matrix, the DES process frees EA and disrupts EA aggregates, entrapping it within the amorphous lignin, greatly enhancing dissolution kinetics in aqueous media.

Moreover, the antioxidant activity of EA-hazelnut-lignin4@PDLLA/PVP and EA-chestnut-lignin2@PDLLA/PVP mats was determined by the CUPRAC assay at the highest concentration previously evaluated for EA-lignin@PDLLA/PVP (0.0025 mg/mL) over the same incubation time (60 min). A lower antioxidant activity was recorded for these samples (A_450_ 0.569 ± 0.002 for EA-chestnut-lignin4@PDLLA/PVP and 0.44475 ± 0.001 for EA-hazelnut-lignin4@PDLLA/PVP), as expected based on the EA release observed (Figure S13). Overall, these data confirm the superior antioxidant performance of the EA-lignin@PDLLA/PVP mats.

### 3.7. Antioxidant Properties of the Released Compounds from EA-Lignin4@PDLLA/PVP

Based on the release results, the antioxidant properties of the compounds eluted from the EA-lignin4@PDLLA/PVP mats were investigated. The release medium obtained after 24 h of incubation in PBS (1×, 37 °C) was collected, dried under vacuum, and redissolved in methanol at known concentrations. Finally, the scavenging capacity of the released solutions was tested by ABTS assay. The antioxidant capacity was expressed as EC_50_, defined as the concentration required to reduce the initial radical concentration by 50%. The released sample exhibited an EC_50_ value of 0.70 ± 0.01 μM, expressed in terms of EA concentration calculated based on the amount of EA released from the e-spun mat. To determine whether this antioxidant capacity was mainly due to the released ellagic acid or to possible traces of lignin, the same assay was performed using a standard solution of EA. The EC_50_ value obtained for pure EA (0.62 ± 0.01 μM) was comparable to that measured for the released sample. These results indicate that the observed antioxidant activity is primarily attributable to EA, with no significant contribution from lignin, likely due to the poor solubility of lignin in aqueous medium. These results demonstrate that the PDLLA/PVP matrix effectively modulates the hydrophilicity and permeability of the e-spun mats, enabling detectable EA release. The data suggest that partial dissolution of PVP in aqueous medium generates micro-porosity within the fibrous scaffold, facilitating water diffusion and subsequent release of EA. This strategy therefore represents an effective approach to tune release behavior in hydrophobic electrospun polyesters while preserving structural integrity and avoiding immediate matrix dissolution as observed for PVP-only films.

### 3.8. Evaluation of Cytocompatibility of the E-Spun Mats

The potential application for regenerative medicine and tissue engineering purposes of the nanofibrous mats was first assessed by testing their cytocompatibility using mesenchymal stem cells (MSCs) and normal human dermal fibroblasts (NDHF). The former are known to orchestrate the in situ activation of the inflammatory cascade, thus representing a suitable model to investigate such properties of diverse materials while the latter are a representative soft tissue cell model. Both cell populations are commonly used to test the in vitro cytotoxicity of biomedical devices to support their use for tissue regeneration purposes in a clinical setting. Concomitantly, the addition of chestnut wood fiber, which contains compounds known for their antioxidant properties, might represent an added value to these mats, in counteracting excessive oxidative stress, which can be detrimental during the first stages of tissue healing. Nonetheless, its addition in the herein-described biomaterial has never been assessed for cytocompatibility. Indeed, nanofibrous mats not including EA-lignin were considered as a control for our experiments, as PDLLA and PVP are two well-established biocompatible materials for skin wound healing purposes [49,50,51,52]. The potential toxicity of the biomaterials was assessed by directly seeding cells onto the mats and allowing them to adhere and grow (Figure 3 and Figure 4). Throughout the experimental period, statistical differences in cell metabolic activity between the two mats were observed: in particular, NDHF showed a higher metabolic activity compared to MSCs. Regarding the former, statistical differences were observed on day 1 (*p* < 0.01) and day 5 (*p* < 0.01) between the two mats; on the other hand, MSCs showed a difference in metabolic activity only on day 3 (*p* < 0.0001). In both cases, the inclusion of EA-lignin in the mats increased the fluorescent signal, and hence the metabolic activity. Both cell populations showed a proliferation trend, with a first initial increasing phase of growth (day 1 to 3), as cells were able to attach and proliferate, followed by a phase characterized by a lower rate of growth (day 3 to 5) in both cell types. The increase in proliferation might be attributed to the presence of EA, which is known to be cytocompatible and elicit cell proliferation [51,52]. These results were also corroborated by fluorescent microscopy imaging showing a visual increase in the number of cells from day 1 to day 5, growing attached to the mats and showing their typical morphology up to day 5 (i.e., a central cellular body with different branches). Based on these results, no signs of toxicity were noted, and experiments concerning their antioxidant potential were consequently performed.

### 3.9. Cytoprotective Effects of EA-Lignin Mats in an Oxidative Stress Environment

Oxidative stress is involved in different physiological processes, such as skin aging, and pathophysiological conditions, such as diabetes, neurodegenerative diseases, or cancer. Particularly, high levels of reactive oxygen species (ROS) can lead to impaired tissue repair, resulting, for example, in delayed skin wound healing or complications such as the generation of a chronic wound or hard-to-heal ulcer [53,54]. For these reasons, in biomedical research, different strategies to attenuate oxidative stress have been widely investigated, and potential antioxidant activity has been found especially in natural products, which are rich in bioactive compounds [55,56]. For inducing a status of oxidative stress, NDHF and MSCs were exposed to hydrogen peroxide (H_2_O_2_), a well-known ROS that can cause oxidative stress by directly damaging cells (e.g., inducing lipid peroxidation and/or DNA damage) and further promoting the production of ROS (indirectly, such as hydroxyl radicals) [57]. After 2 h, cellular distress was observed as the addition of H_2_O_2_ induced a decrease in cell viability to 50% compared to cells grown in standard conditions (i.e., cells grown onto plastic) (Figure 5A,B). However, when cells were seeded onto both types of mats, cell viability was slightly recovered, especially in treated mats (Figure 5A,B). As a matter of fact, the presence of EA-lignin resulted in a significant increase in cell metabolic activity (around 75%) compared to mats without EA-lignin (Figure 5); nonetheless, basal levels of metabolic activity were not fully recovered in NDHF (Figure 5A). On the other hand, MSCs were shown to possess a higher resistance to excessive levels of H_2_O_2_ as cells showed levels of metabolic activity similar to control conditions (Figure 5B). This result aligns with the well-documented endogenous redox machinery of MSCs, which might be attributed to the higher basal activity of antioxidant enzymes (such as superoxide dismutase, catalase, or glutathione peroxidase) compared to somatic cells [58,59]. In contrast, NDHFs possess lower baseline levels of antioxidant enzymes, making them intrinsically more susceptible to acute oxidative stress, lipid peroxidation, and loss of viability in high ROS environments [60,61]. These differential responses highlight the different biological uses of EA-lignin nanofibrous mats for potential biomedical applications such as skin tissue engineering. For NDHFs, EA could provide an essential exogenous scavenging support that helps protect and increase cell survival during the functional activity of cells [60,62], whereas for MSCs, the mat could act as an antioxidant scaffold serving as a cytoprotective shield against extracellular oxidative stress, reducing cell damage and strain on their redox defense mechanisms and supporting cells in maintaining their functional state [57].

Furthermore, the scavenging activity of treated mats was tested by quantifying ROS levels using a commercial fluorescent probe. The presence of EA-lignin (and of EA expectedly released in cell culture medium) significantly lowered the presence of ROS, thus accounting for the higher metabolic activity in both cell populations (Figure 5C,D). Our findings are in line with the literature reports showing EA as a potent scavenging agent in dermal fibroblasts in inflammatory settings [52,63,64,65,66].

## 4. Conclusions

In conclusion, functional mats were successfully prepared by electrospinning poly(d,l--lactide)/poly(vinylpyrrolidone) (PDLLA/PVP) loaded with an ellagic acid (EA)-enriched lignin at 2% and 4% *w*/*w*. The EA-lignin was easily recovered through a sustainable, eco-friendly protocol from CWF by use of a DES. Morphological and chemical characterization of the obtained mats confirmed that this strategy led to a successfully incorporation of EA-lignin, effectively overcoming the typical processing limitations of this phenolic biopolymer. Remarkably, the hybrid mats demonstrated outstanding, dose-dependent antioxidant capacity as evaluated by ABTS and CUPRAC assays, with the EA-lignin4@PDLLA/PVP scaffold showing the most prominent performance. The EA-lignin4@PDLLA/PVP mats also provided a highly sustained and controlled delivery of bioactive EA in physiological conditions. More importantly, in vitro experiments confirmed that the electrospun mats possess excellent cytocompatibility toward both normal human dermal fibroblasts (NHDFs) and mesenchymal stem cells (MSCs). When exposed to hydrogen-peroxide-induced oxidative insults, the EA-lignin-loaded scaffolds effectively preserved cell metabolic activity and significantly mitigated intracellular ROS accumulation, providing strong redox protection. Overall, these results demonstrate that these hybrid mats—particularly the 4% *w*/*w* EA-lignin loaded scaffold—represent a highly promising, eco-friendly, and bioactive electrospun device for biomedical applications.

## Figures and Tables

**Figure 1:**
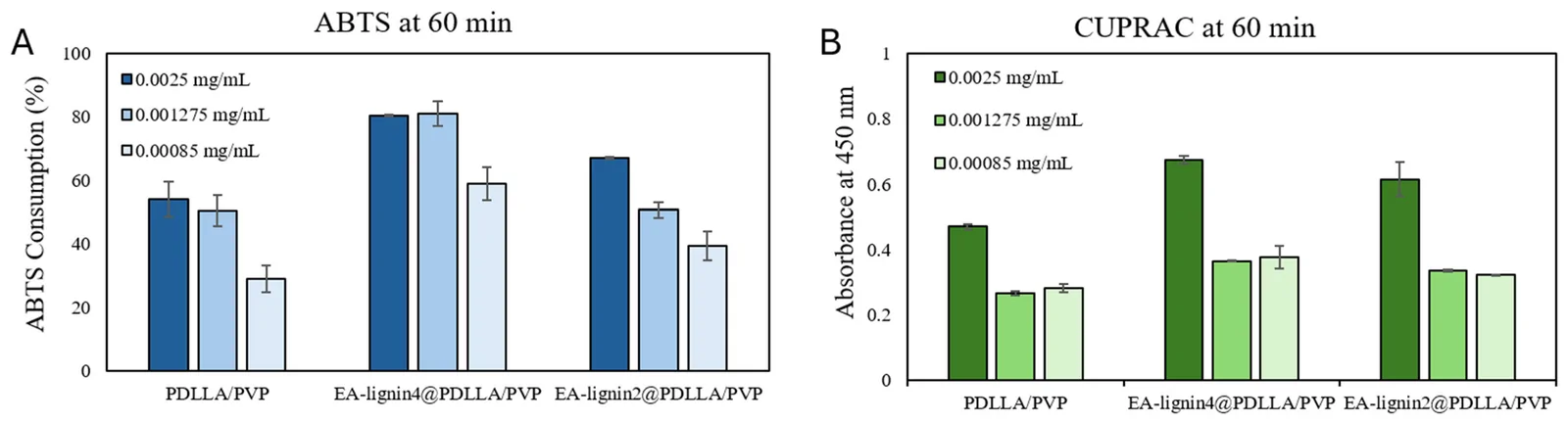
Comparative antioxidant capacity of PDLLA/PVP, EA-lignin4@PDLLA/PVP, and EA-lignin2@PDLLA/PVP formulations after 60 min of incubation at three different concentrations (0.0025, 0.001275, and 0.00085 mg/mL): (**A**) radical scavenging activity evaluated via ABTS assay, expressed as ABTS consumption (%); (**B**) reducing power evaluated via CUPRAC assay, expressed as absorbance at 450 nm. Data represent the mean ± standard deviation (SD).

**Figure 2:**
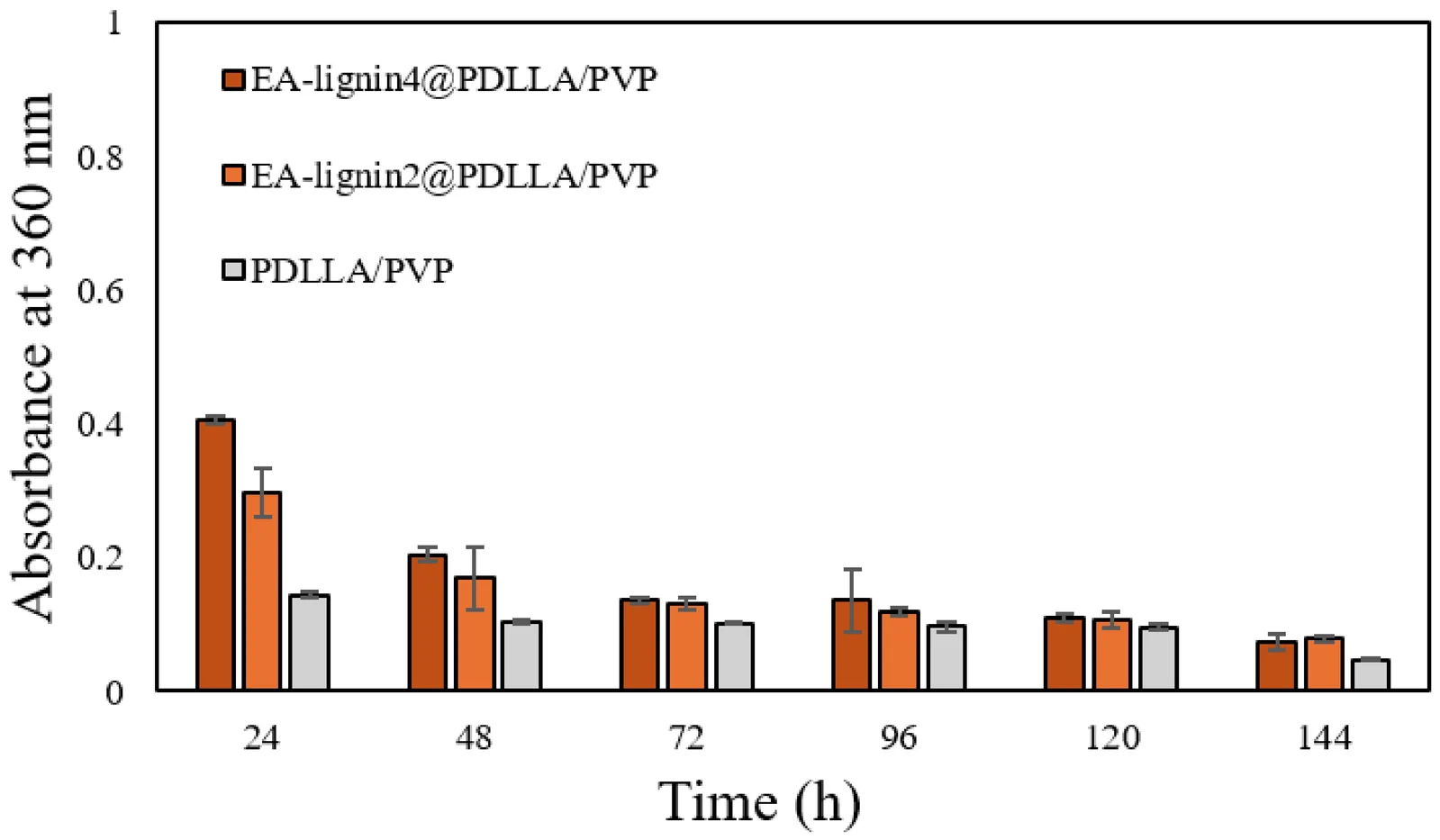
Absorbance at 360 nm of the release medium from EA-lignin@PDLLA/PVP mats at different loadings and from pure PDLLA/PVP mats, with PBS refreshed every 24 h over a 6 d period.

**Figure 3:**
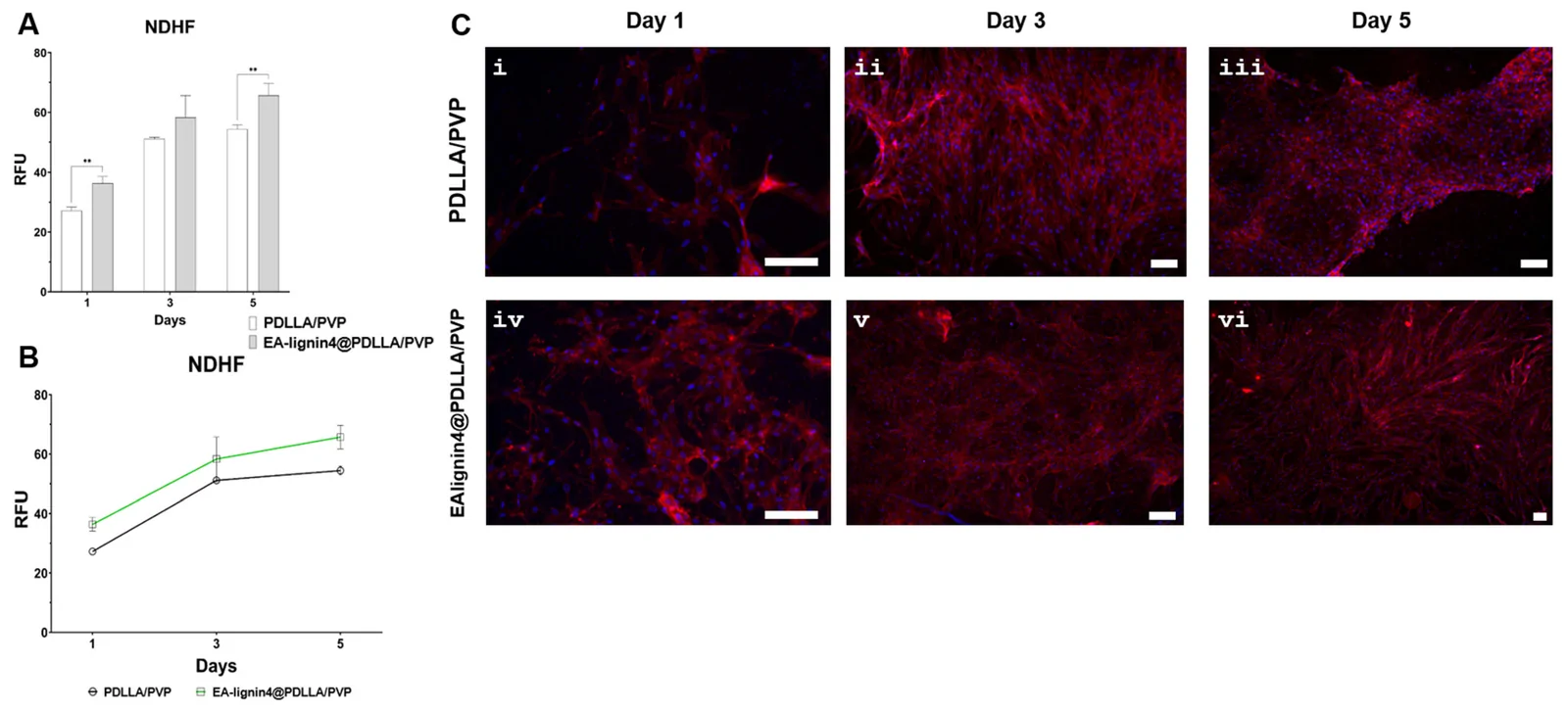
(**A**) The metabolic activity of NDHF grown on PDLLA/PVP or EA-lignin (EA-lignin4@PDLLA/PVP) mats was evaluated at 1, 3, and 5 d in culture; (**B**) metabolic activity over time; (**C**) Overlay of representative images stained for f-actin filaments (phalloidin; red) and nuclei (Hoechst 33342; blue) of NDHF grown onto mats with (**i**–**iii**) or without EA-enrinched lignin (**iv**–**vi**) at day 1 (**i**,**iv**), day 3 (**ii**,**v**), and day 5 (**iii**,**vi**) showing the typical morphology of fibroblasts; scale bar = 100 μm; data are expressed as mean ± SEM; ** *p* < 0.01.

**Figure 4:**
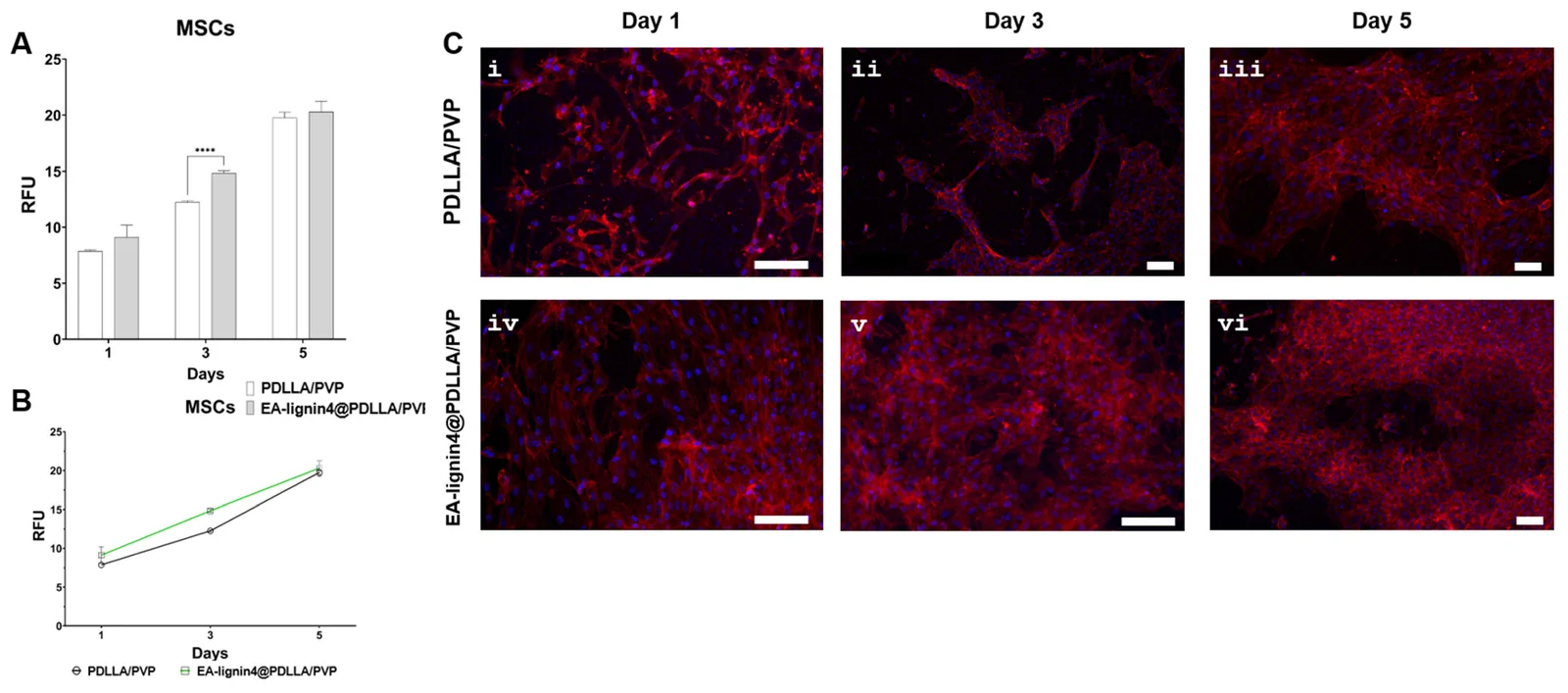
(**A**) The metabolic activity of MSCs grown on PDLLA/PVP or EA-lignin (EA-lignin4@PDLLA/PVP) mats evaluated after 1, 3, and 5 d in culture; (**B**) metabolic activity over time; (**C**) Overlay of representative images stained for f-actin filaments (phalloidin; red) and nuclei (Hoechst 33342; blue) of MSCs grown onto mats with (**i**–**iii**) or without EA-enrinched lignin (**iv**–**vi**) at day 1 (**i**,**iv**), day 3 (**ii**,**v**), and day 5 (**iii**,**vi**) showing the typical morphology of fibroblasts; scale bar = 100 μm; data are expressed as mean ± SEM; **** *p* < 0.0001.

**Figure 5:**
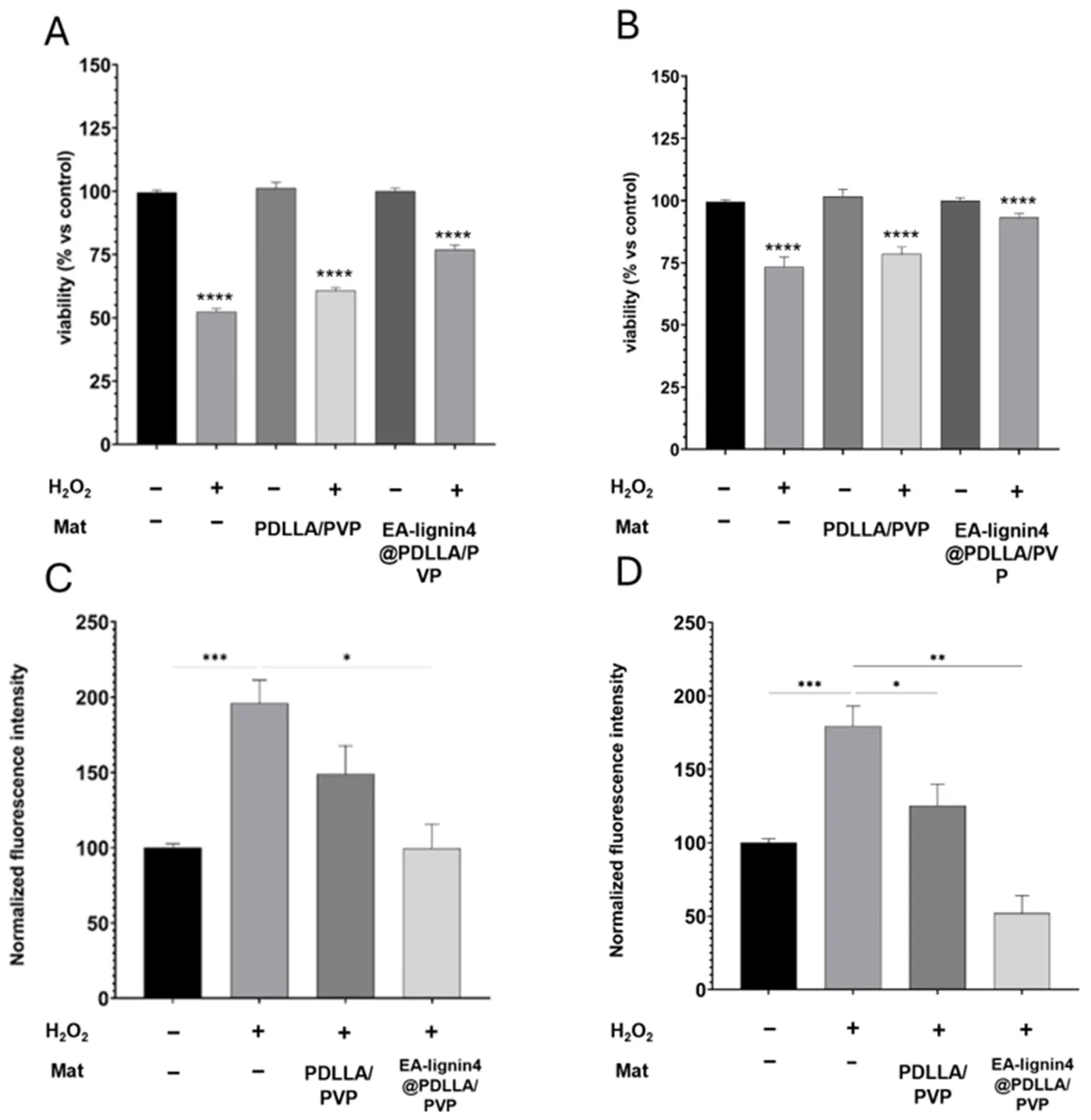
In order to induce oxidative stress, NDHF (**A**,**C**) and MSCs (**B**,**D**) were exposed to 200 μM H_2_O_2_ for 2 h on PDLLA/PVP and EA-lignin4@PDLLA/PVP plastic mats. The metabolic activity of (**A**) NDHF and (**B**) MSCs was tested along with the quantification of intracellular ROS in (**C**) NDHF and (**D**) MSCs. (**A**,**B**) Viability is expressed as % of the respective control condition without H_2_O_2_; (**C**,**D**) ROS levels were normalized on the baseline data (i.e., cells grown in standard conditions). Data are expressed as mean ± SEM; * *p* < 0.05, ** *p* < 0.01, *** *p* < 0.001, **** *p* < 0.0001.

**Table 1:** Electrospinning samples composition ^a^.

Scaffold	PDLLA ^b^ (% *w*/*v*)	PVP ^c^ (% *w*/*w*)	EA-Lignin ^c^ (% *w*/*w*)
EA-lignin2@PDLLA/PVP	10	15	2
EA-lignin4@PDLLA/PVP	10	15	4
EA-hazelnut-lignin4@PDLLA/PVP	10	15	4
EA-chestnut-lignin4@PDLLA/PVP	10	15	4
**PDLLA/PVP**	10	15	**-**
**PDLLA**	10	**-**	**-**

^a^ control samples are in bold; ^b^ weight by the overall volume; ^c^ weight with respect to PDLLA.

**Table 2:** Characteristic frequencies for the e-spun mats and proposed assignments.

Frequency (cm^−1^)						
EA- Lignin2 @PDLLA/PVP	EA- Lignin4 @PDLLA/PVP	PDLLA/ PVP	PDLLA	EA-Lignin Powder	PVP Powder	Proposed Assignment
-	-	3700–3100	-	3550, 3500–3090	3643–3128	ν (O-H)
2995–2945	2996–2945	2998–2948	2996–2945	2916–2849	3018–2807	ν (aliphatic C-H)
1753 (ester)	1755 (ester)	1754 (ester)	1753 (ester)	1732 (ester)	1656–1651 (lactam)	ν (C=O)
1717 (EA-lignin)	1717 (EA-lignin)	1658 (lactam)		1715 (ketone)		
1652 (lactam)	1652 (lactam)			1700 (conj. aldehyde)		
-	-	-	-	1614–1582, 1510	-	ν (aromatic C=C)
1453	1456–1447	1474–1429	1453	1451	1492–1421	δ (aliphatic C-H)

**Table 3:** SWCA values reported as mean value ± standard deviation (SD) (n = 3).

Scaffold	SWCA (°)
EA-lignin2@PDLLA/PVP	98 ± 1
EA-lignin4@PDLLA/PVP	141 ± 1
PDLLA/PVP	75 ± 1
PDLLA	115 ± 2

**Table 4:** SEM image analysis results showing digital porosity, apparent *Rq,* and fiber diameter values reported as mean value ± standard deviation (SD) (n = 3 for DP and apparent *Rq*; n = 150 for fiber diameter analysis).

Scaffold	DP (%)	*Rq* (AU)	Fiber Diameter (μm)
EA-lignin2@PDLLA/PVP	53.8 ± 3.4	0.49 ± 0.03	0.43 ± 0.10
EA-lignin4@PDLLA/PVP	52.3 ± 6.6	0.53 ± 0.04	0.44 ± 0.07
PDLLA/PVP	28.3 ± 5.0	0.48 ± 0.02	0.40 ± 0.08
PDLLA	29.9 ± 7.6	0.56 ± 0.07	0.37 ± 0.07

## Data Availability

The original contributions presented in this study are included in the Supplementary Material. Further inquiries can be directed to the corresponding author.

## Supplementary Materials

The following supporting information can be downloaded at https://www.mdpi.com/article/10.3390/antiox15101224/s1, Figure S1: SEM images of e-spun mats of PDLLA (A), PDLLA/PVP (B), EA-lignin4@PDLLA/PVP (C), EA-lignin2@PDLLA/PVP (D). Figure S2: ATR FT-IR spectrum of PDLLA/PVP (red curve), PVP (green curve) and PDLLA (black curve). FT-IR bands are designated by colored dots (see text for explanation). Figure S3: ATR-FTIR spectrum of ellagic-acid-enriched lignin as powder. FT-IR bands are designated by colored dots (see text for explanation). Figure S4: A. ATR FT-IR spectrum of EA-lignin2@PDLLA/PVP (magenta curve), EA-lignin4@PDLLA/PVP (blue curve), PDLLA/PVP (red curve), PDLLA (black curve). B. Magnified region from 1000 to 1800 cm^−1^. Figure S5: SWCA measurements on the electrospun mats A (EA-lignin2@PDLLA/PVP), B (EA-lignin4@PDLLA/PVP), C (PDLLA/PVP), and D (PDLLA). Figure S6: Fiber diameter distribution for EA-lignin2@PDLLA/PVP (A), EA-lignin4@PDLLA/PVP (B), PDLLA/PVP (C), and PDLLA (D). The red curves represent the Gaussian fits. Figure S7: Average fiber diameter values (± standard deviation) of electrospun scaffolds (*n* = 150). Figure S8: Evaluation of the antioxidant properties using ABTS assay for PDLLA/PVP, EA-lignin2@PDLLA/PVP, and EA-lignin4@PDLLA/PVP samples. The graphs illustrate the percentage of free radical consumption (%) as a function of incubation time (20, 40, and 60 min) evaluated at three distinct sample concentrations: A. 0.0025 mg/mL, B. 0.001275 mg/mL, and C. 0.00085 mg/mL. Data are presented as mean values ± standard deviation (SD). Figure S9: Evaluation of the antioxidant capacity using CUPRAC assay for PDLLA/PVP, EA-lignin2@PDLLA/PVP, and EA-lignin4@PDLLA/PVP samples. The graphs report the absorbance at 450 nm as a function of incubation time (20, 40, and 60 min) evaluated at three distinct sample concentrations: A. 0.0025 mg/mL, B. 0.001275 mg/mL, and C. 0.00085 mg/mL. Data are presented as mean values ± standard deviation (SD). Figure S10: Cumulative absorbance at 360 nm over 144 h following the release from EA-lignin@PDLLA/PVP mats at different loadings and from PDLLA/PVP mats. Figure S11: UV–Vis spectrum of the solution collected after 24 h of incubation in PBS (1×) at 37 °C of PDLLA/PVP and EA-lignin4@PDLLA/PVP and, for comparison, of pure EA solution (C_f_ 0.013 mM) and EA-lignin (C_f_ 0.002 mg/mL) in PBS (1x). Figure S12: SEM images of the e-spun mats EA-lignin2@PDLLA/PVP (A), EA-lignin4@PDLLA/PVP (B), PDLLA/PVP (C), and PDLLA (D) after 6 days of incubation in PBS. Figure S13: Absorbance at 360 nm of the release medium from EA-hazelnut-lignin4@PDLLA/PVP and EA-chestnut-lignin4@PDLLA/PVP mats, with PBS refreshed every 24 h over a 6 d period.

## Abbreviations

The following abbreviations are used in this manuscript:

ABTS	2,2′-azino-bis(3-ethylbenzothiazoline-6-sulfonic acid)
ATR-FTIR	Attenuated Total Reflectance–Fourier Transform Infrared Spectroscopy
BOAS	Bead-on-a-String
CUPRAC	Cupric Reducing Antioxidant Capacity
CWF	Chestnut Wood Fiber
DES	Deep Eutectic Solvent
DMEM	Dulbecco’s Modified Eagle Medium
DMSO	Dimethyl Sulfoxide
DNA	Deoxyribonucleic Acid
EA	Ellagic Acid
ECM	Extracellular Cellular Matrix
EDTA	Ethylenediaminetetraacetic Acid
FBS	Fetal Bovine Serum
H_2_O_2_	Hydrogen Peroxide
HFP	1,1,1,3,3,3-Hexafluoro-2-propanol
HPLC	High-Performance Liquid Chromatography
KCl	Potassium Chloride
MSCs	Mesenchymal Stem Cells
NHDF	Normal Human Dermal Fibroblasts
PBS	Phosphate-Buffered Saline
PDLLA	Poly(D,L-lactide)
PLLA	Poly(L-lactide)
PVP	Poly(vinylpyrrolidone)
ROS	Reactive Oxygen Species
SEM	Scanning Electron Microscopy
SWCA	Static Water Contact Angle
TRITC	Tetramethylrhodamine B Isothiocyanate
UV-Vis	Ultraviolet–Visible Spectroscopy
WCA	Water Contact Angle
