# Supplementary material for: Incorporation of Ellagic-Acid-Enriched Lignin into Hydrophilic Electrospun PDLLA/PVP Fibrous Scaffolds Imparts Remarkable Antioxidant and Cellular Redox Protection Properties
Source: Antioxidants (Basel). 2026 Sep 22;15(10):1224. doi: 10.3390/antiox15101224 (PMC13700913; doi:10.3390/antiox15101224)
Supplement: Supplementary file 1 [file antioxidants-15-01224-s001.zip › antioxidants-4482014-supplementary.pdf]

Incorporation of Ellagic Acid–Enriched Lignin into Hydrophilic Electrospun PDLLA/PVP Fibrous Scaffolds Imparts Remarkable Antioxidant and Cellular Redox Protection Properties

Rita Argenziano [1], Antonio Laezza [2,3], Luca Melotti [4,5], Roberta Sacchetto [4], Brigida Bochicchio [2], Antonietta Pepe [2], Samuele Giovando [6], Lucia Panzella [1] and Alessandra Napolitano [1,*]

[1] Department of Chemical Sciences, University of Naples "Federico II", Via Cintia 4, 80126 Naples, Italy; rita.argenziano@unina.it (R.A.); panzella@unina.it (L.P.); alesnapo@unina.it (A.N.)

[2] Department of Basic and Applied Sciences (DISBA), University of Basilicata, Via dell'Ateneo Lucano 10, 85100 Potenza, Italy; brigida.bochicchio@unibas.it (B.B.); antonietta.pepe@unibas.it (A.P.); antonio.laezza@unibas.it (A.L.)

[3] Department of Theoretical and Applied Sciences (DiSTA), eCampus University, Via Isimbardi 10, 22060 Novedrate, Italy; antonio.laezza@uniecampus.it (A.L.)

[4] Department of Comparative Biomedicine and Food Science (BCA), University of Padova, Viale dell'Università 16, 35020 Legnaro, Italy; luca.melotti@unipd.it (L.M.); roberta.sacchetto@unipd.it (R.S.)

[5] Department of Human Sciences, Link Campus University, Via del Casale di San Pio V 44, 00165 Rome, Italy; l.melotti@unilink.it (L.M.)

[6] Centro Ricerche per la Chimica Fine Srl for Silvateam Spa, Via Torre 7, 12080 San Michele Mondovì, (Cuneo), Italy; sgiovando@silvateam.com

* Correspondence: alesnapo@unina.it; Tel.: +39-081-674133

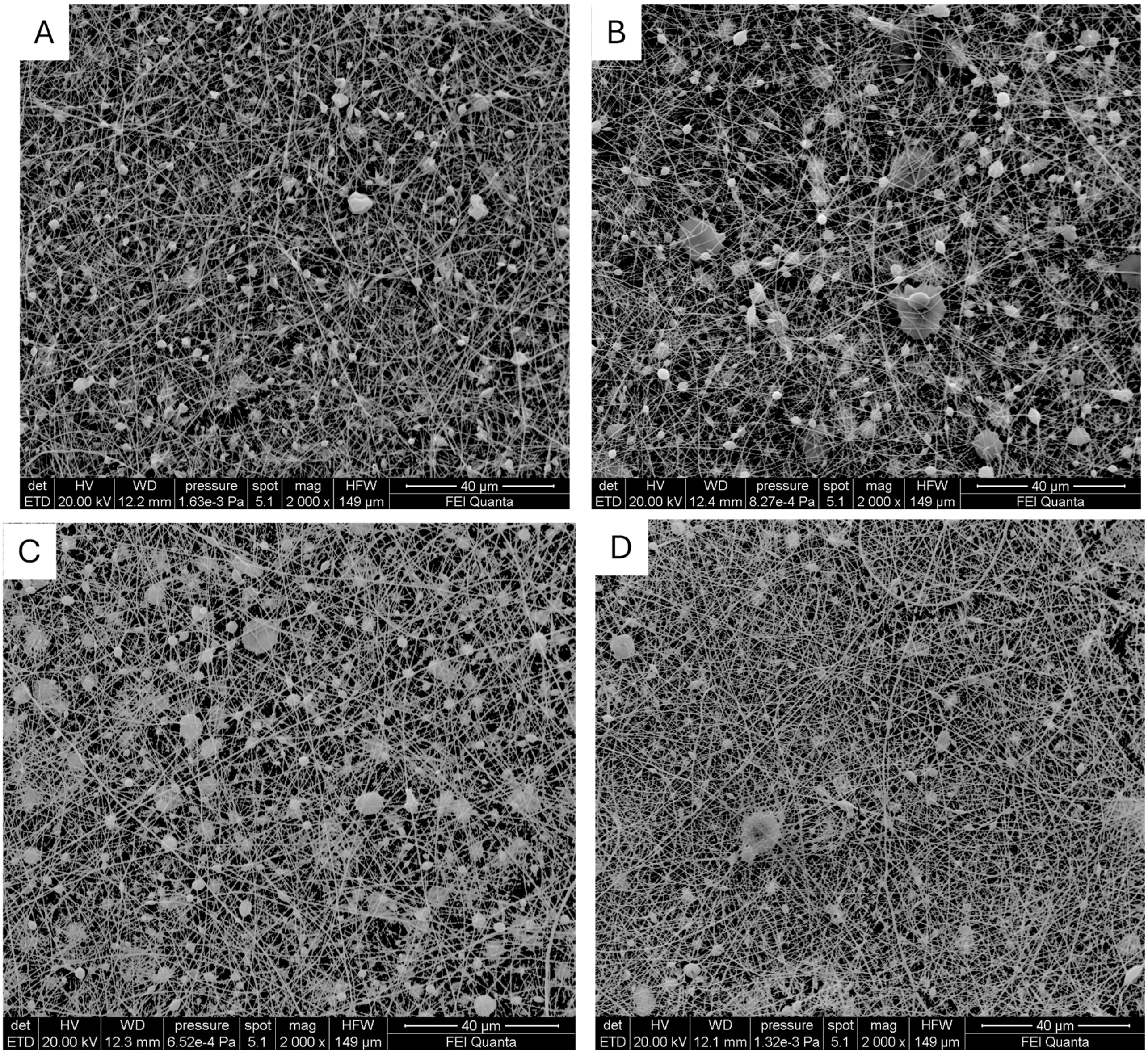

Figure S1: SEM images of e-spun mats of PDLLA (A), PDLLA/PVP (B), EA-lignin4@PDLLA/PVP (C), EA-lignin2@PDLLA/PVP (D).

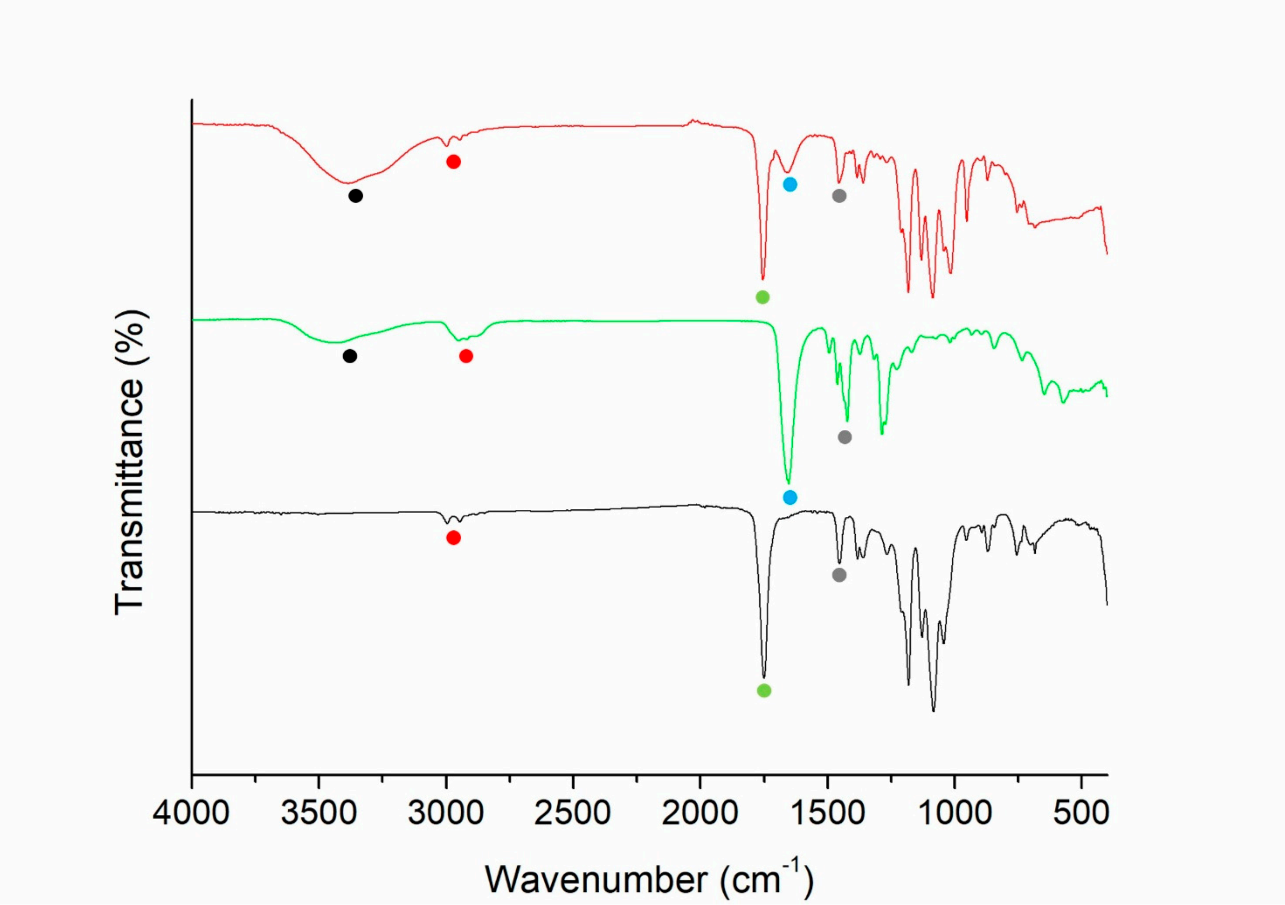

Figure S2: ATR FT-IR spectrum of PDLLA/PVP (red curve), PVP (green curve) and PDLLA (black curve). FT-IR bands are designated by colored dots (see text for explanation).

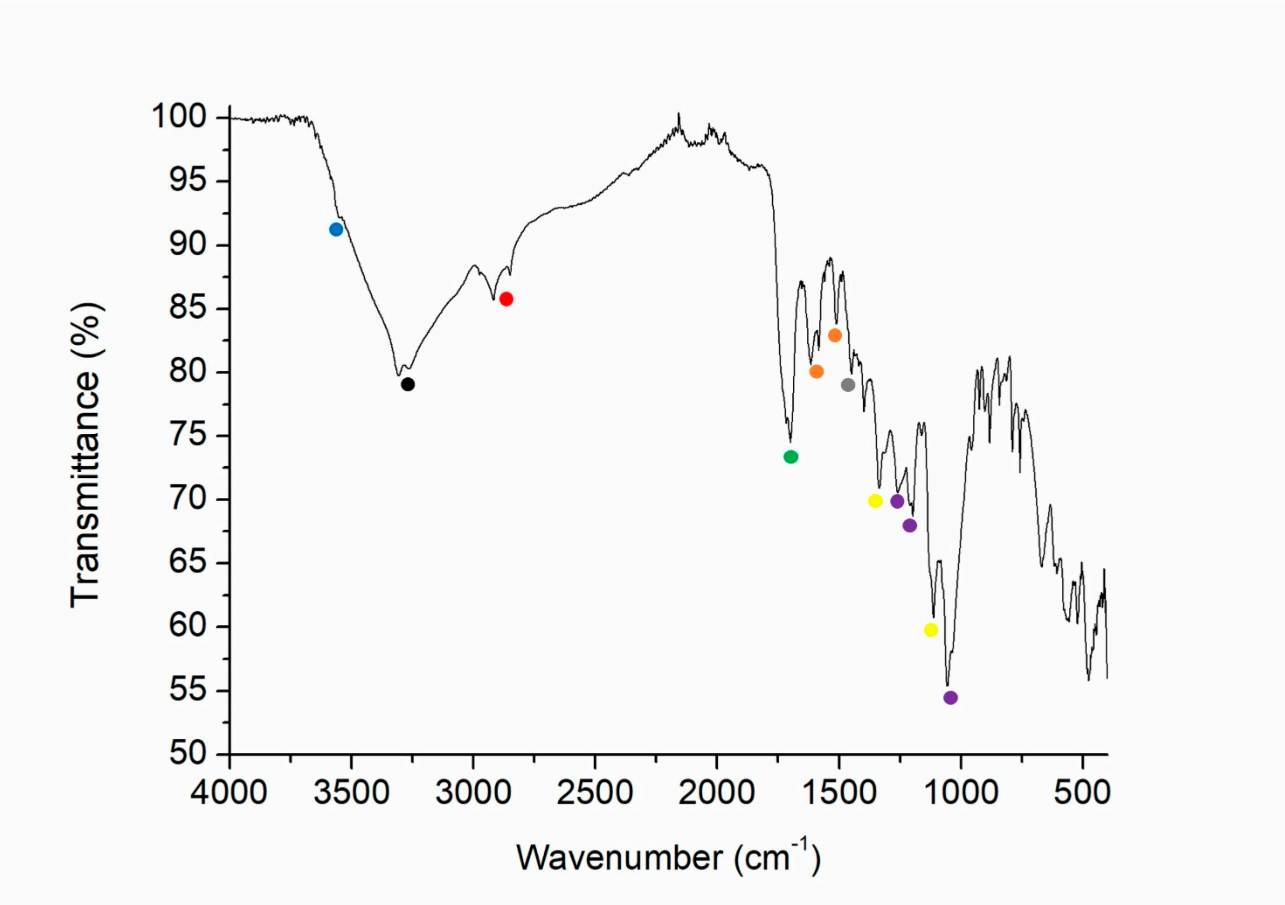

Figure S3: ATR-FTIR spectrum of ellagic acid enriched lignin as powder. FT-IR bands are designated by colored dots (see text for explanation).

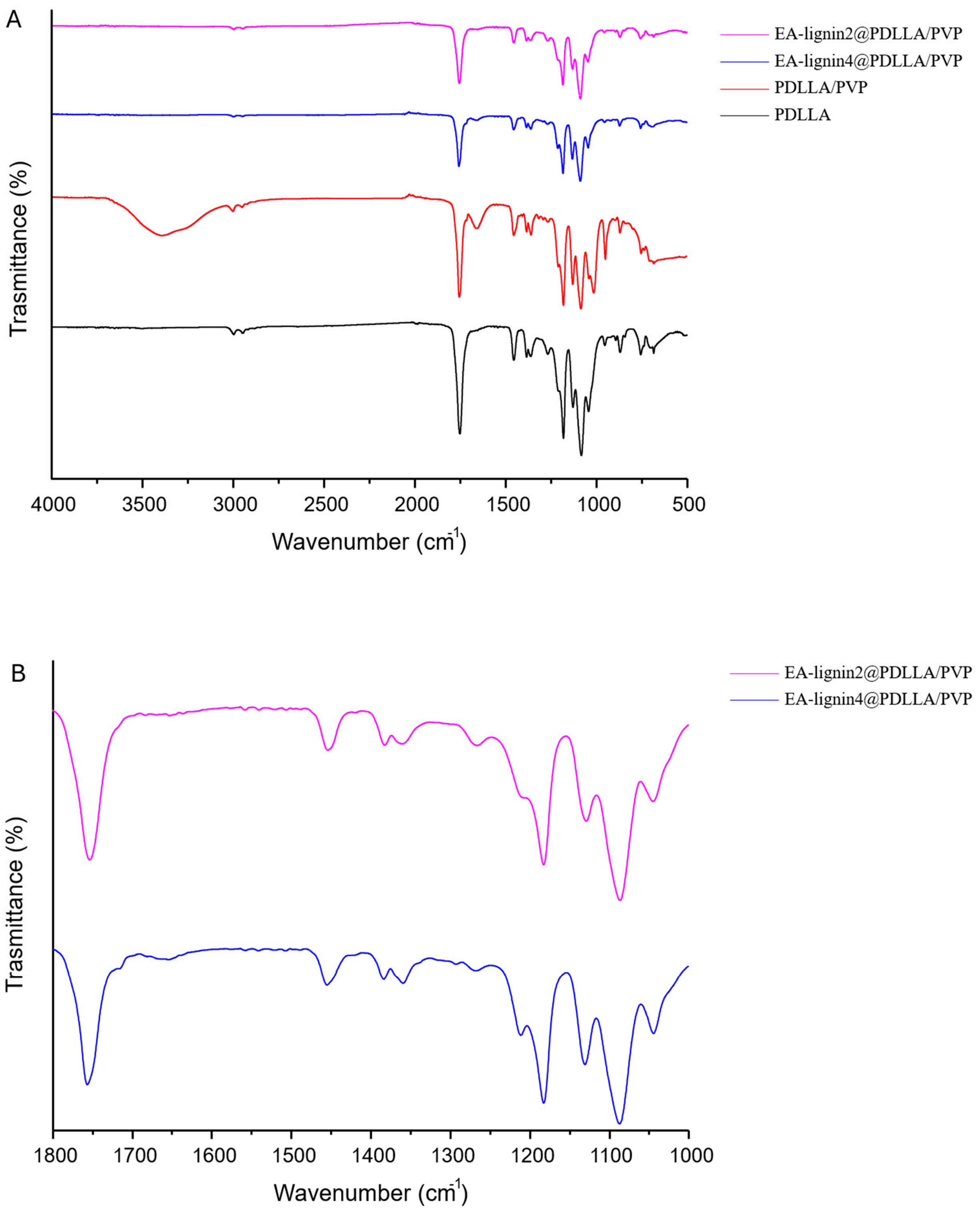

Figure S4: A. ATR FT-IR spectrum of EA-lignin2@PDLLA/PVP (magenta curve), EA-lignin4@PDLLA/PVP (blue curve), PDLLA/PVP (red curve), PDLLA (black curve). B. Magnified region from 1000 to 1800 cm^{-1}

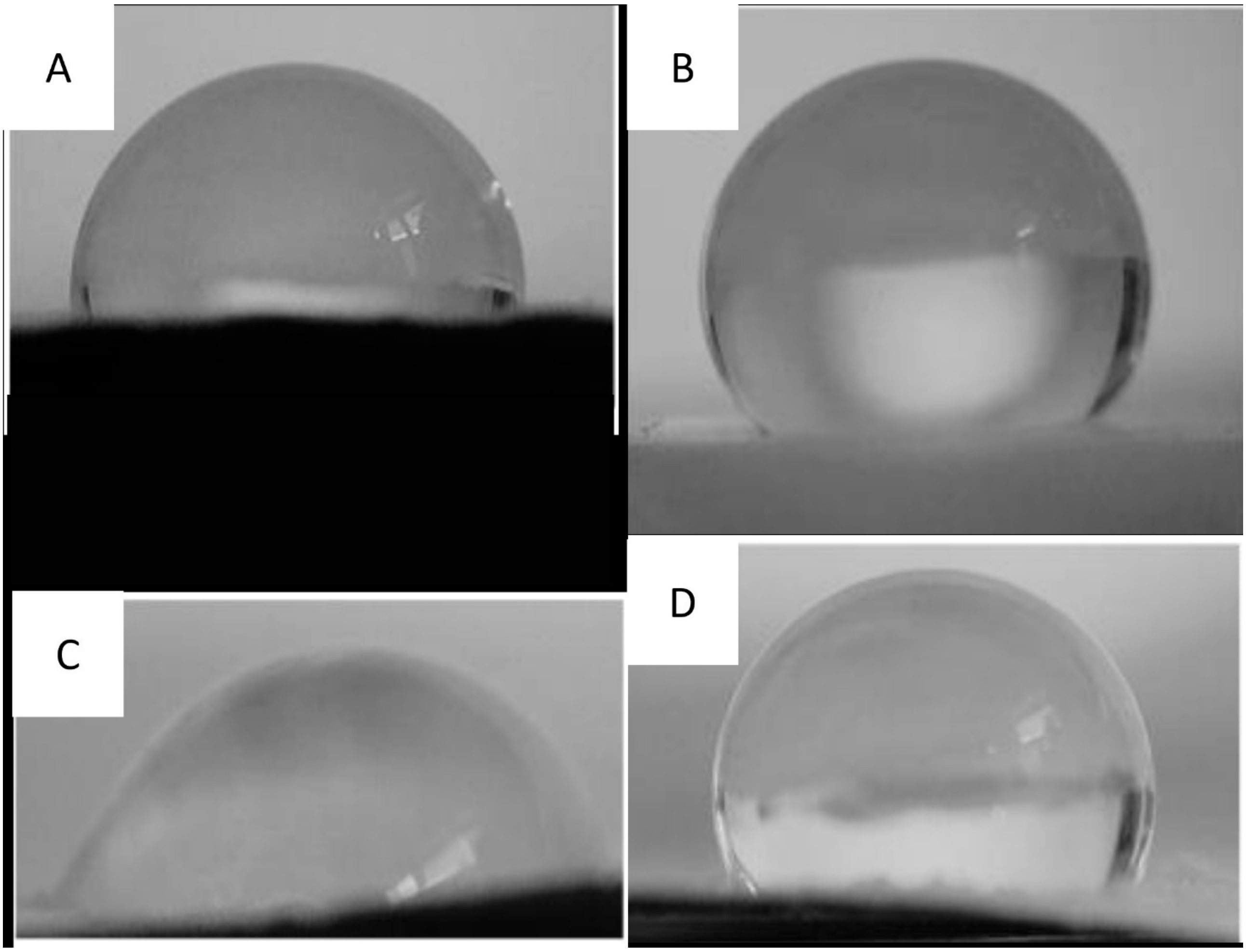

Figure S5: SWCA measurements on the e-spun mats EA-lignin2@PDLLA/PVP (A), EA-lignin4@PDLLA/PVP (B), PDLLA/PVP (C), and PDLLA (D).

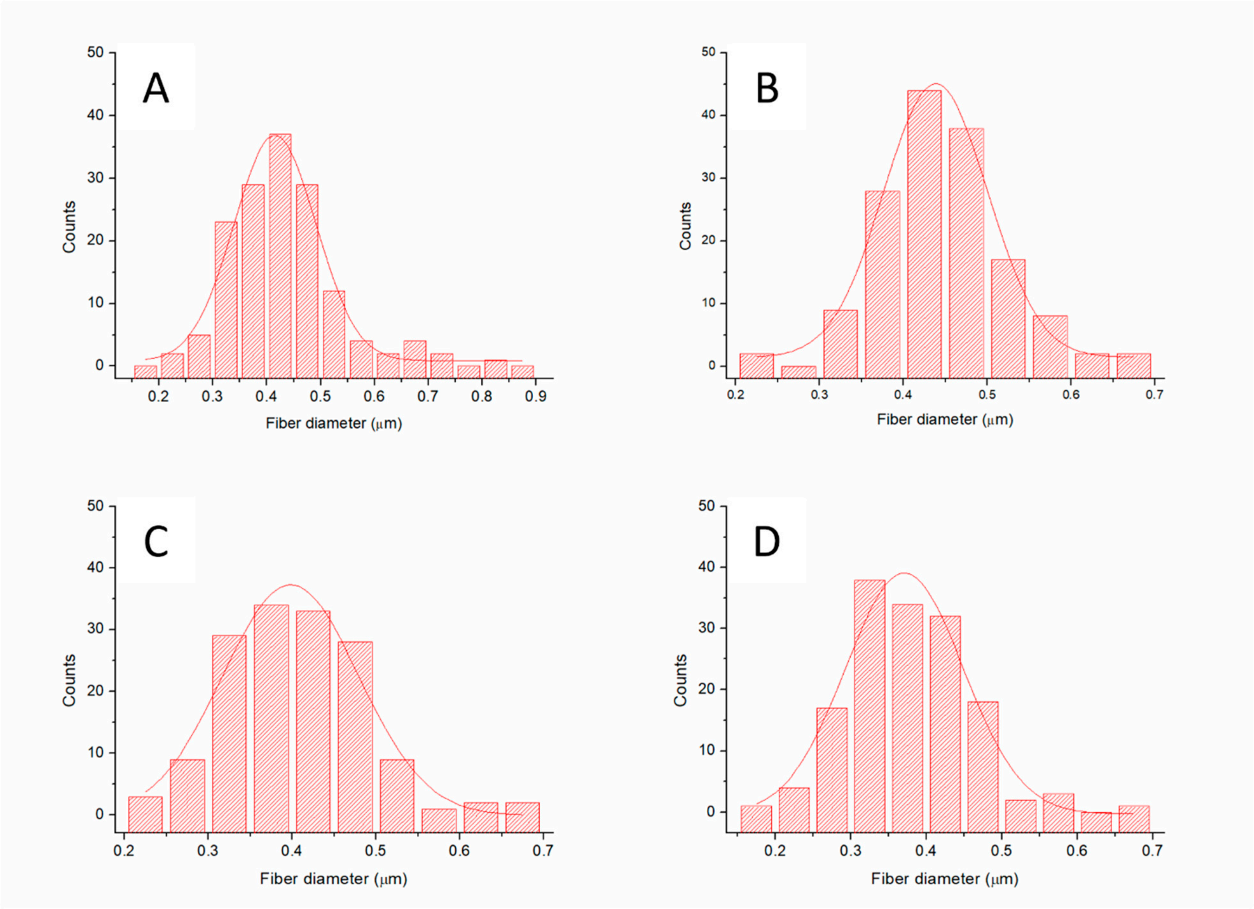

Figure S6: Fiber diameter distribution for EA-lignin2@PDLLA/PVP (A), EA-lignin4@PDLLA/PVP (B), PDLLA/PVP (C), and PDLLA (D). The red curves represent the Gaussian fits

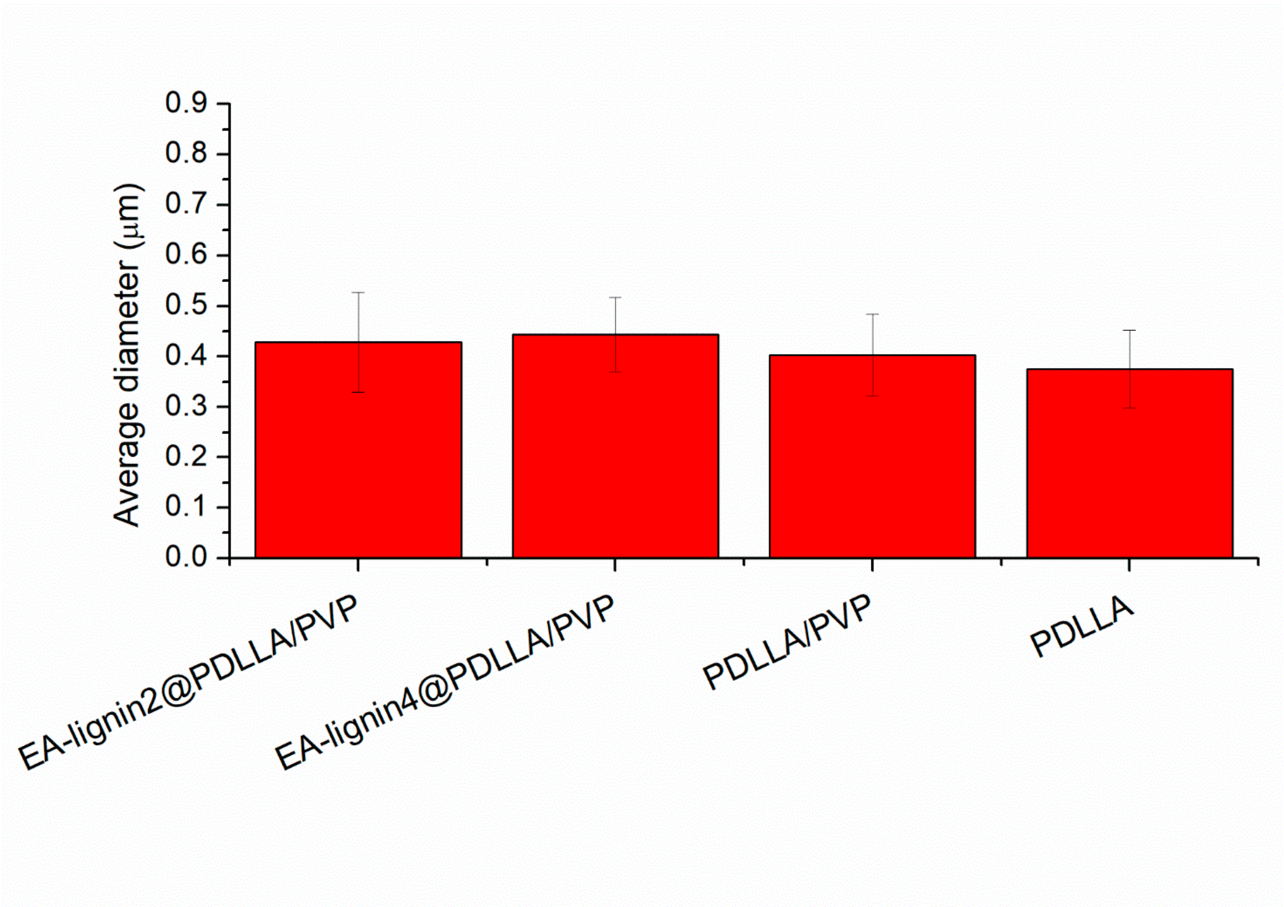

Figure S7: Average fiber diameter values (± standard deviation) of electrospun scaffolds (*n* =150)

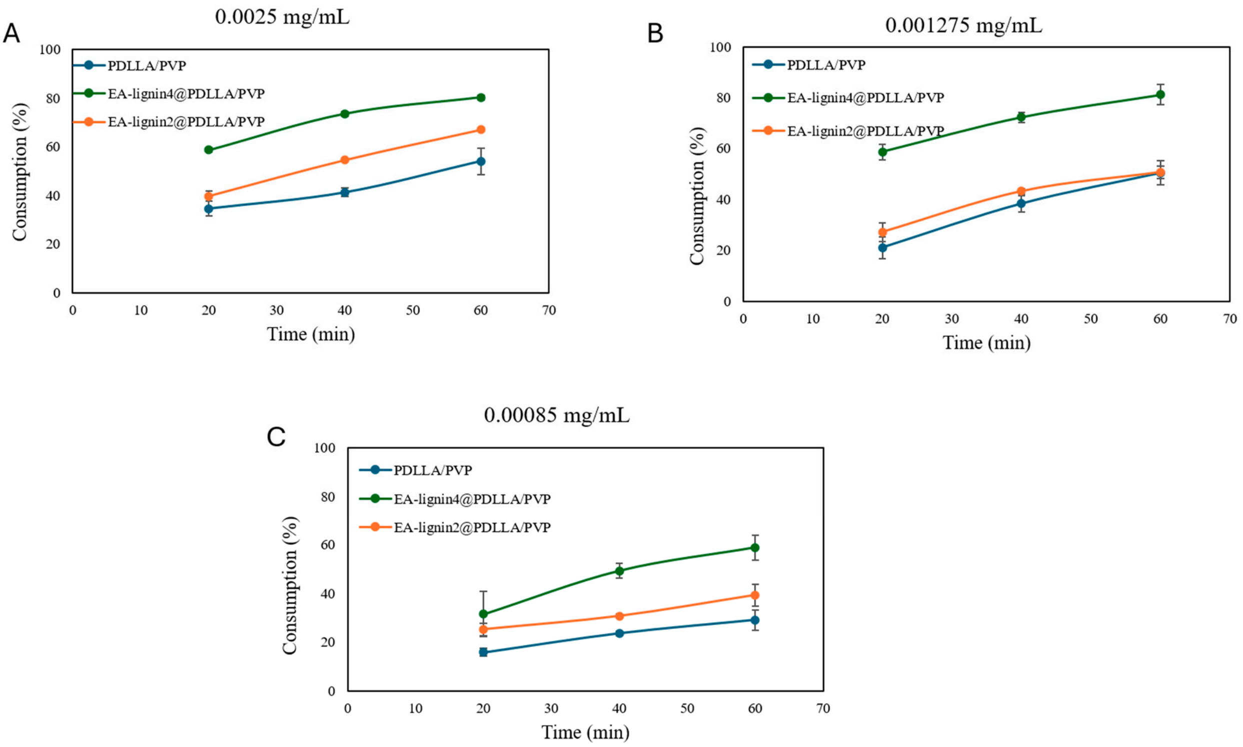

Figure S8. Evaluation of the antioxidant properties using ABTS assay for PDLLA/PVP, EA-lignin2@PDLLA/PVP, and EA-lignin4@PDLLA/PVP samples. The graphs illustrate the percentage of free radical consumption (%) as a function of incubation time (20, 40, and 60 min) evaluated at three distinct sample concentrations: A. 0.0025 mg/mL, B. 0.001275 mg/mL, and C. 0.00085 mg/mL. Data are presented as mean values ± standard deviation (SD).

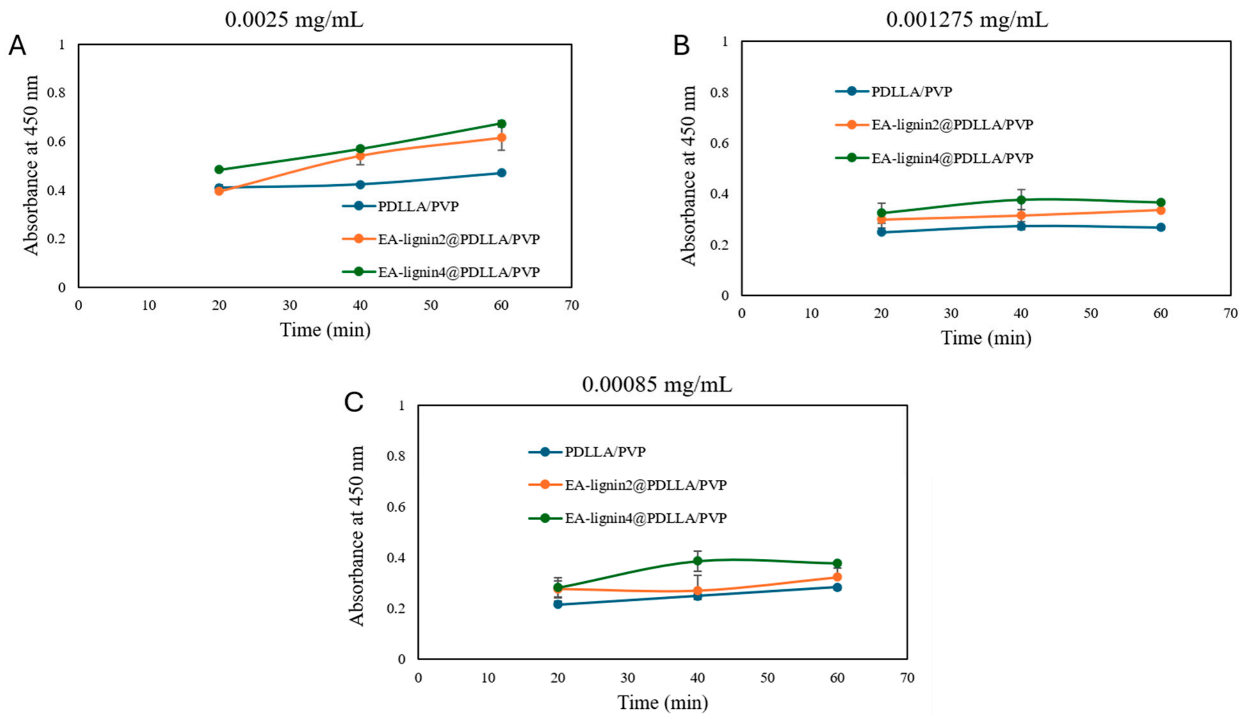

Figure S9. Evaluation of the antioxidant capacity using CUPRAC assay for PDLLA/PVP, EA-lignin2@PDLLA/PVP, and EA-lignin4@PDLLA/PVP samples. The graphs report the absorbance at 450 nm as a function of incubation time (20, 40, and 60 min) evaluated at three distinct sample concentrations: A. 0.0025 mg/mL, B. 0.001275 mg/mL, and C. 0.00085 mg/mL. Data are presented as mean values ± standard deviation (SD).

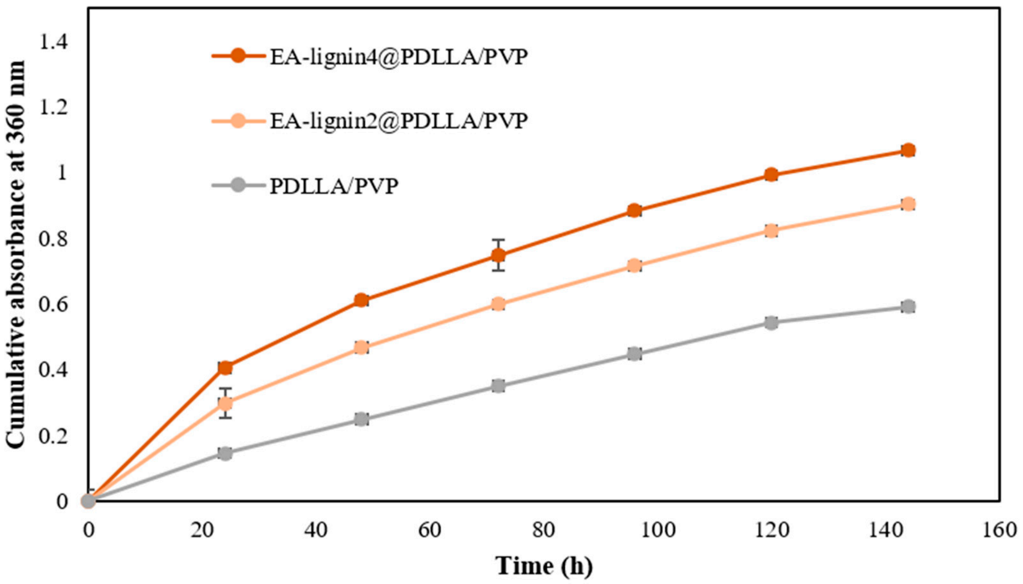

Figure S10. Cumulative absorbance at 360 nm over 144 h following the release from EA-lignin@PDLLA/PVP mats at different loadings and from PDLLA/PVP mats.

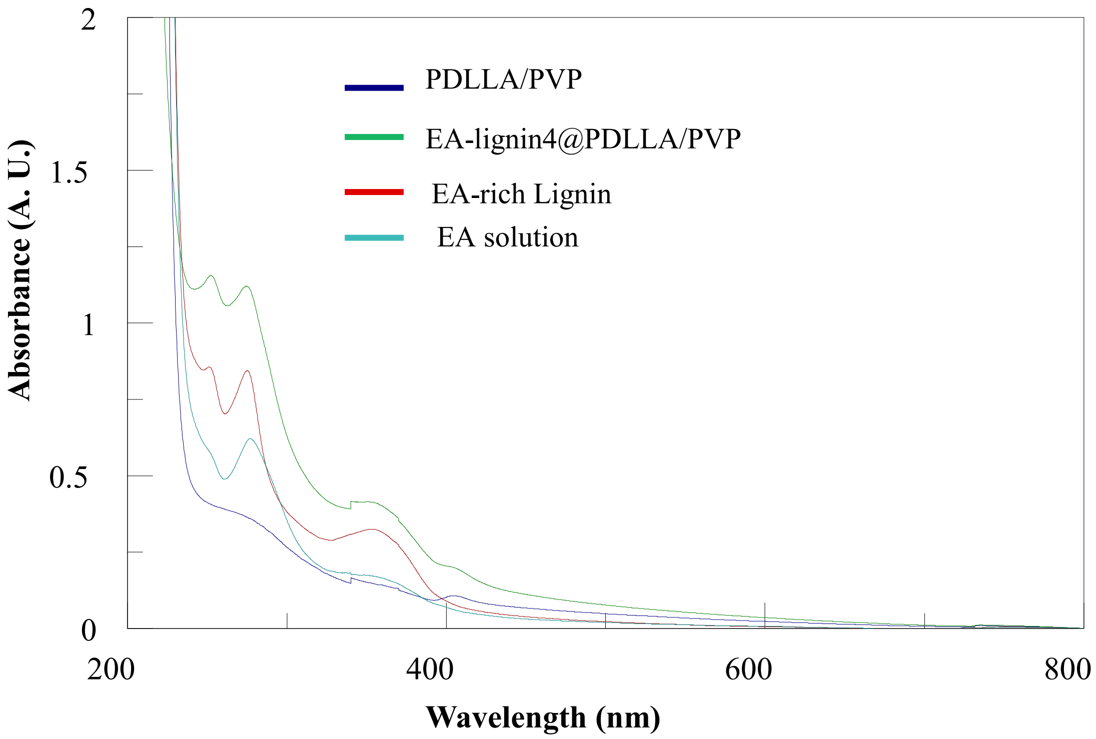

Figure S11. UV–Vis spectrum of the solution collected after 24 h of incubation in PBS (1×) at 37 °C of PDLLA/PVP and EA-lignin4@PDLLA/PVP and, for comparison, of pure EA solution (C_f 0.013 mM) and EA-lignin (C_f 0.002 mg/mL) in PBS (1x).

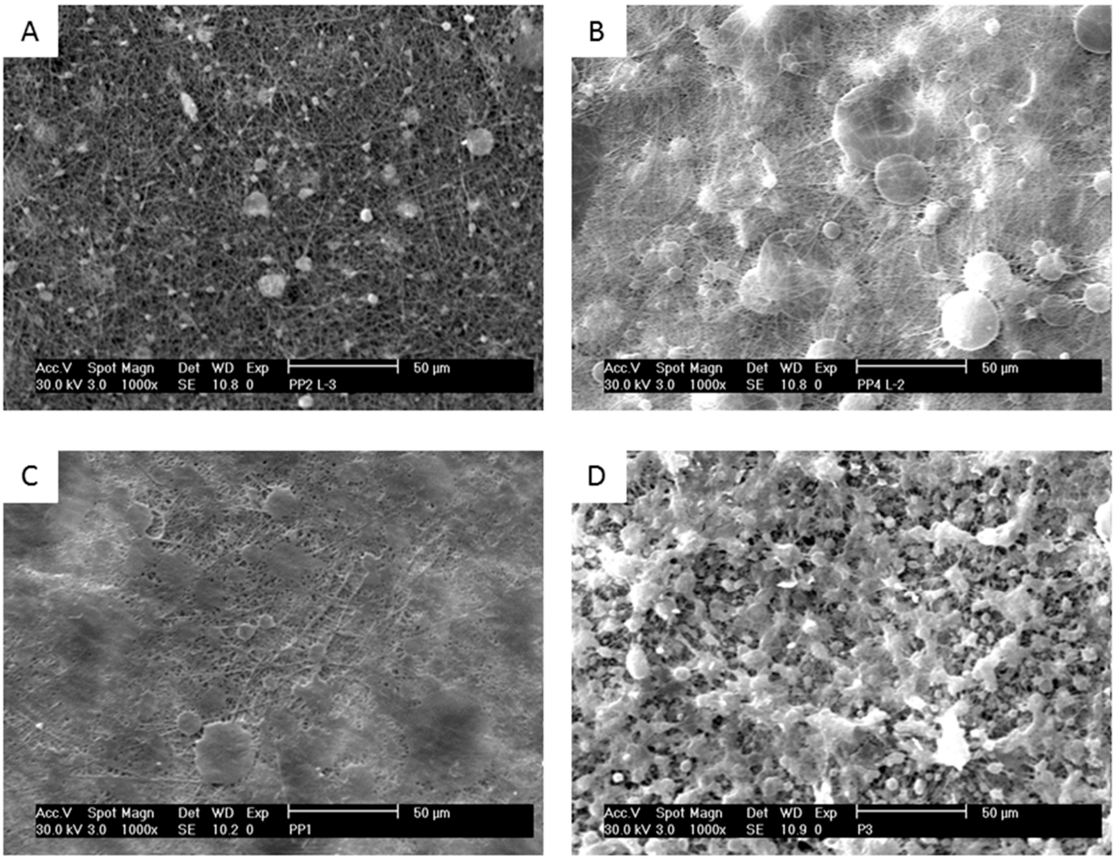

Figure S12: SEM images on the e-spun mats EA-lignin2@PDLLA/PVP (A), EA-lignin4@PDLLA/PVP (B), PDLLA/PVP (C), and PDLLA (D) after 6 days of incubation in PBS.

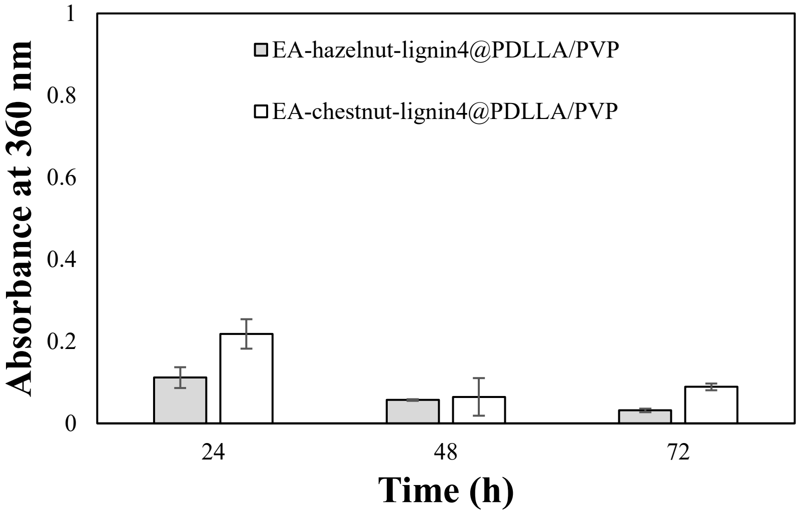

Figure S13: Absorbance at 360 nm of the release medium from EA-hazelnut-lignin4@PDLLA/PVP and EA-chestnut-lignin4@PDLLA/PVP mats, with PBS refreshing every 24 h over a 6-d period.
